# A Comparative Species Framework to Identify Candidate Salivary miRNAs Associated with Breast Cancer Risk

**DOI:** 10.3390/ijms27146198

**Published:** 2026-07-11

**Authors:** James L. Miller, Mariza DaCosta, Kimaya M. Bakhle, Lisa Lai, Dawn E. Post, Rebecca M. Harman, Gerlinde R. Van de Walle

**Affiliations:** 1 Baker Institute for Animal Health, College of Veterinary Medicine, Cornell University, Ithaca, NY 14853, USA; james.miller@jax.org (J.L.M.); md160@rice.edu (M.D.); kmb368@cornell.edu (K.M.B.); rmh12@cornell.edu (R.M.H.); 2 Department of Surgery, SUNY Upstate Medical University, Syracuse, NY 13210, USA; lail@upstate.edu; 3 Biorepository Core and Department of Urology, Syracuse, NY 13210, USA; postd@upstate.edu; 4 The Roslin Institute and Royal (Dick), School of Veterinary Studies, University of Edinburgh, Midlothian EH25 9RG, UK

**Keywords:** breast cancer, microRNAs, biomarkers, species-to-species variation, mammary epithelial cells

## Abstract

Accurate assessment of early indicators of breast cancer (BC) is critical to improve detection strategies and patient prognosis. Our research group takes a comparative species approach to study early BC detection by capitalizing on data from species with inherently low or high incidence of mammary cancer to supplement rare human samples. Circulating microRNAs (c-miRNAs) are a promising class of molecules that have the potential to serve as biomarkers for BC risk and disease detection. The expression of these non-coding RNAs controls numerous cellular processes and their dysregulation is associated with various pathological conditions, including cancer. To explore whether c-miRNA expression profiles can identify individuals with early-stage BC, we conducted a multi-species pilot study. We analyzed biofluid samples (i.e., saliva, serum, and plasma) from patients with early-stage BC and patients with no prior history of cancer and mammosphere-derived epithelial cells (MDECs) from dogs (canines) and horses (equines), species with a relatively high and low incidence of mammary cancer, respectively. We identified 16 candidate c-miRNAs that were upregulated in saliva from patients in the early-stage BC group when compared to the control patient group. Notably, 6 of these c-miRNAs (i.e., miR-361, miR-148b, miR-205, miR-186, miR-223, and miR-197) were also found to be secreted at higher levels by canine MDECs when compared to equine MDECs. Although individual and combinatorial assessment of these six c-miRNAs in a larger human cohort did not confirm their potential association with early breast cancer detection, the data in this study do introduce a novel comparative framework in which species-to-species variation in cancer susceptibility may inform the identification of candidate biomarkers for human disease.

## 1. Introduction

Breast cancer (BC) is the most frequently diagnosed malignancy among women and remains the second leading cause of cancer-related mortality worldwide [1,2,3]. The American Cancer Society estimated that in 2022, approximately 287,850 new cases of invasive breast cancer and 43,250 deaths occurred among U.S. women [1]. Global data from GLOBOCAN similarly show over 2.3 million new cases and 685,000 deaths in 2020, accounting for nearly 12% of all cancers diagnosed that year [2,3]. Incidence continues to increase annually by approximately 1%, with the sharpest rise observed among premenopausal women [1]. Despite substantial advances in therapy, prognosis remains highly dependent on the stage at diagnosis—five-year survival exceeds 90% for localized disease but declines to roughly 30% once distant metastases are present [1,4].

Conventional screening tools such as mammography, ultrasound, and magnetic resonance imaging (MRI) detect existing lesions rather than early molecular changes (Supplementary Figure S1). Mammography, though the current standard of care, has limited sensitivity in dense breast tissue and carries a risk of false positives and overdiagnosis. MRI provides improved sensitivity but is costly and not broadly accessible [5]. These shortcomings highlight the need for additional, minimally invasive screening strategies capable of identifying individuals at elevated risk before tumors become clinically detectable, especially as such early screening expands preventative and therapeutic options (Supplementary Figure S1).

Several statistical models have been developed to predict BC risk, including the Gail, Tyrer–Cuzick, and BOADICEA models [6,7,8]. Each integrates hereditary, hormonal, and lifestyle variables, yet they often fail to predict most cases because roughly 85% of breast cancers arise sporadically in women without strong family history or pathogenic variants [9]. Moreover, these models rely on static demographic and genetic information rather than dynamic molecular changes that precede malignancy. This limitation underscores the value of circulating biomarkers that can capture real-time molecular states of breast tissue.

Circulating microRNAs (c-miRNAs) have emerged as particularly promising biomarker candidates. miRNAs are small, non-coding RNAs that regulate gene expression post-transcriptionally and are released into biofluids within exosomes or protein complexes, conferring remarkable stability [10,11]. Because they can be measured in serum, plasma, urine, and saliva, c-miRNAs provide an avenue for repeated, non-invasive molecular assessment. Multiple studies have documented dysregulated c-miRNAs in plasma and serum of breast-cancer patients when compared to control patients [12,13,14] and salivary miRNA profiles have been associated with several malignancies, including breast, oral, and pancreatic cancers [15,16,17]. Saliva represents an especially advantageous biofluid for molecular screening as it is simple to collect, requires no specialized personnel, and allows longitudinal monitoring with minimal discomfort. In BC specifically, upregulation of miR-21, miR-200c, and miR-27b in saliva has been reported in patient cohorts and was shown to reflect molecular events within the mammary epithelium [16,18]. Because saliva also contains tumor-derived exosomal cargo, it can mirror systemic disease states [19]. Therefore, salivary c-miRNAs can offer a non-invasive approach to detect molecular signatures of early tumorigenesis or premalignant cellular changes that may not yet be visible radiographically.

Species-to-species comparative oncology provides a powerful complementary strategy for identifying and validating candidate biomarkers. Domestic dogs (canines) and horses (equines) display a markedly different susceptibility to developing mammary cancer, presenting a natural contrast that can elucidate mechanisms of risk and resistance. Mammary tumors are among the most common neoplasms in intact female dogs, accounting for nearly half of all spontaneous tumors, and they share key histopathologic and molecular features with human BC, including hormone-receptor status, molecular-subtype distribution, and metastatic behavior [20,21,22]. In contrast, mammary tumors in horses are extremely rare, but when they do occur, reports describe similar hormone-receptor profiles and histological organization to human tumors [23,24,25]. These interspecies differences in mammary cancer incidence, combined with cross-species similarities in tumor biology, present the dog as a valuable high BC risk proxy and the horse as a low BC risk proxy. Since miRNAs are highly structurally conserved across species, they represent a class of promising biomarkers that may have shared etiopathological roles across mammals. Comparative analyses of miRNA expression in mammary cell secretions between these two species may help prioritize candidates with conserved regulatory roles in mammary oncogenesis that can potentially predict BC risk in humans.

In this study, we applied a comparative species approach to identify candidate c-miRNAs potentially associated with early BC presence. By integrating canine (high-risk) and equine (low-risk) mammary cell miRNA profiles with human salivary miRNA data, we aimed to pinpoint conserved miRNAs that may serve as early indicators of mammary epithelial transformation, or susceptibility to transformation. This framework leverages naturally occurring biological diversity to filter for functionally relevant biomarkers and focuses on saliva as a minimally invasive biofluid suited for repeated screening. Together, these innovations may help refine BC assessment, improve personalized screening strategies, and expand opportunities for earlier intervention in women at risk.

## 2. Results

### 2.1. Study Population for a Pilot Analysis of Human Samples

Nanostring nCounter analysis was used for initial assessment of circulating (c)-miRNA in samples from a small discovery pilot group of female breast cancer (BC) patients (*n* = 3) that were selected based on hormone receptor (HR) expression profiles—estrogen receptor (ER), progesterone receptor (PR), and human epidermal growth factor receptor 2 (HER2)—and on tumor grade—grade 1–4. Selected samples came from patients diagnosed with grade 1, ER+/PR+/HER2− luminal-like invasive carcinomas, which are typically representative of early-stage disease and with luminal-like subtypes being the most prevalent molecular forms of BC, comprising approximately 50–70% of cases [26,27]. Furthermore, luminal-like carcinomas of comparable histopathology to humans are frequently observed in canine models [22], making the analysis of patients with luminal-like tumors particularly relevant within a comparative species framework. Although grade 1 ER+/PR+/HER2− tumors are generally associated with lower biological risk than higher-grade tumors, patients with these tumors are typically at an earlier stage of breast cancer progression and remain at increased risk of developing advanced-stage breast cancer compared with the control population described below. Therefore, defining a biomarker panel at this early stage may enable detection of early breast cancer or, potentially, prediction of breast cancer risk before detection by conventional methods. Details regarding patient age, tumor profiles, and pathological findings are summarized in Table 1. None of the patients had a prior diagnosis of BC and genetic testing confirmed the absence of known mutations such as Breast Cancer Associated Genes 1/2 (BRCA1/2) or Partner and Localizer of BRCA2 (PALB2), and other clinically significant variants. These individuals were age-matched with participants (*n* = 3) who had no history of cancer, but were all admitted due to nonmalignant uterine-related pathologies (i.e., pelvic organ prolapse) (Table 1).

### 2.2. Differentially Expressed C-miRNAs in Saliva, Serum and Plasma

The multiplexed Nanostring nCounter platform was used to evaluate differences in c-miRNA expression profiles across saliva, serum and plasma samples from the small discovery pilot patient cohort (Table 1). This technique utilizes a probe-based, amplification-free detection method to quantify 827 annotated miRNAs, minimizing amplification bias associated with cDNA and enabling accurate detection of c-miRNAs directly from biofluids.

All samples passed quality control, with no QC flags reported. Binding densities ranged from 0.14 to 0.20 read/um^2^, falling within the assay’s detection limits [28]. Although each sample contained the same RNA input (100 ng), variations in ligation efficiency were observed among samples, as shown by differences in ligation positive control signals (Supplementary Figure S2a). These discrepancies were addressed by normalizing to ligation positive controls A and B (Supplementary Figure S2b), which corrected for inter-sample variability. No quality control (QC) flags emerged during Nanostring analysis, confirming that the RNA samples were suitable in both quality and quantity for downstream comparisons.

**Figure 1:**
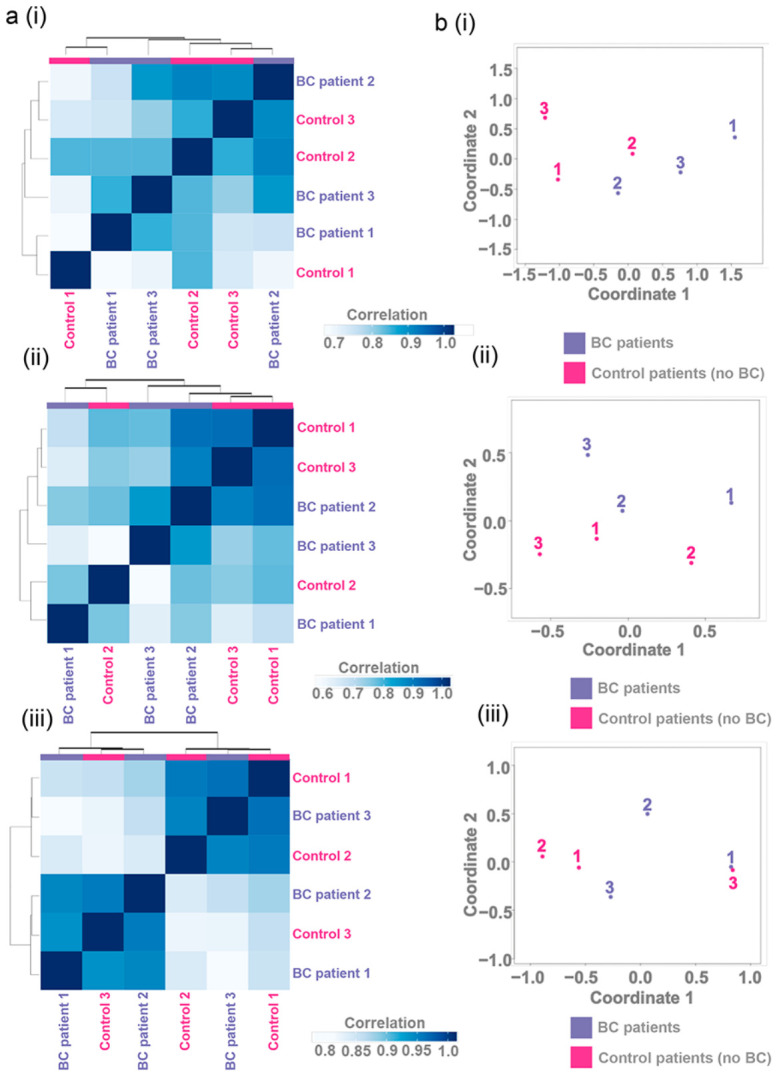
Circulating microRNA (c-miRNA) expression in plasma does not correlate well with breast cancer (BC) status. (**a**). Heatmaps illustrating expression correlations in saliva (**i**), serum (**ii**) and plasma (**iii**) samples. They were generated in the ROSALIND^®^ platform, which computes pairwise Pearson correlation coefficients (R) between all sample pairs and with darker colors indicating greater expression correlation between samples. miRNAs were grouped using Partitioning Around Medoids (PAM); dendrograms on the left and top of the heatmaps indicate predicted clustering patterns with close branching indicating statistical hierarchical clustering. (**b**). Multidimensional scaling (MDS) plots of saliva (**i**), serum (**ii**) and plasma (**iii**) samples, with each point representing an individual sample.

Sample correlation heatmaps (Figure 1a) showed differing levels of clustering within each biofluid group, with plasma samples exhibiting the lowest intra-group correlation (Figure 1a(iii)). Dendrogram clustering (Figure 1a), which separates samples by hierarchical clustering and places them similar samples side-by-side on the heat maps if they show similar expression profiles, demonstrated that plasma samples did not segregate into distinct clusters according to disease status, suggesting heterogeneity in miRNA expression. Multidimensional scaling (MDS) plots demonstrated clear intra-group clustering in saliva (Figure 1b(i)) and serum (Figure 1b(ii)) compared to plasma (Figure 1b(iii)), highlighting greater variability in plasma c-miRNA expression than other biofluids assessed.

**Figure 2:**
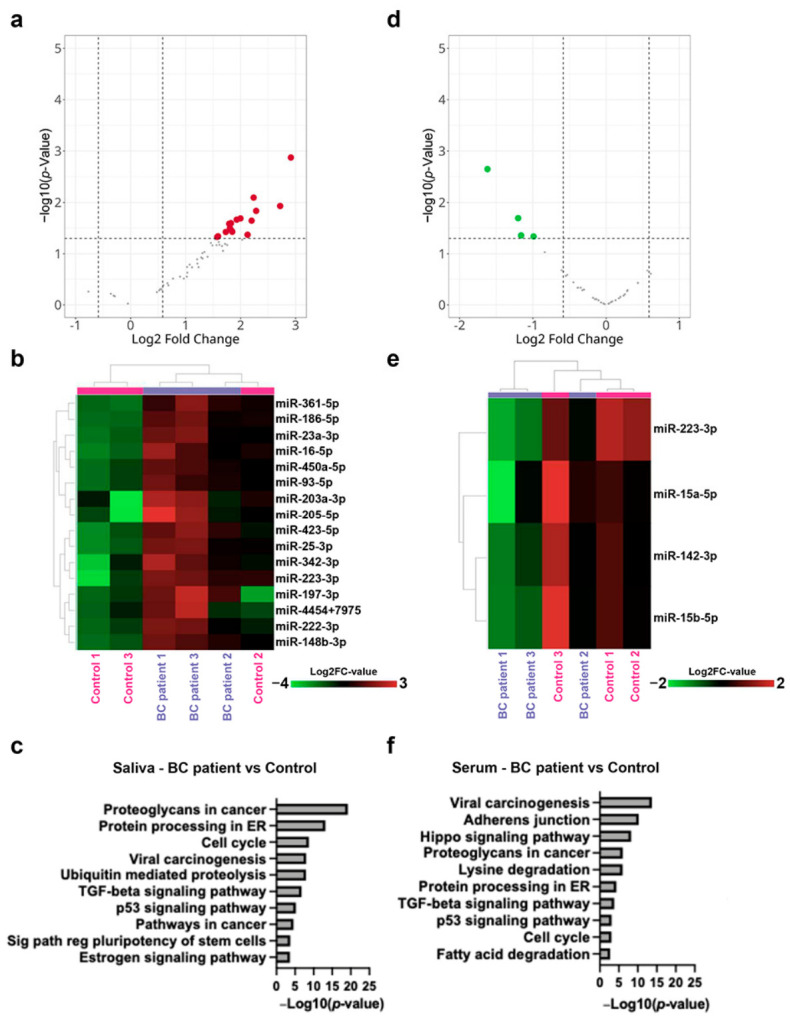
Circulating microRNAs (c-miRNAs) detected in saliva do correlate with breast cancer (BC) status. (**a**). Volcano plot showing c-miRNAs in saliva. Red points represent c-miRNAs that meet the criteria for significantly differential expression (|Log2FC| ≥ 0.58 and *p* ≤ 0.05) between both groups. Grey dots are detected c-miRNAs that did not pass the thresholds for statistically significant expression (|Log2FC| ≤ 0.58 and *p* ≥ 0.05). (**b**). Corresponding heatmap displaying the expression profiles of these differentially expressed c-miRNAs, where color gradients reflect deviations of each sample’s Log2FC from the mean Log2FC across all samples and dendrograms depicting sample statistical clustering. Fold-change and Log2FC values were calculated using the ROSALIND^®^ platform using the *limma* R package. Color gradient indicates Log2FC values ranging from Log2FC = −4 (light green) to Log2FC = 3 (light red). (**c**). KEGG enrichment bar graph displaying 10 selected gene unions associated with the 16 differentially expressed c-miRNAs shown in (**b**). (**d**). Volcano plot showing c-miRNAs in serum, with green points indicating significantly differentially expressed c-miRNAs that meet the same statistical thresholds (|Log2FC| ≤ 0.58 and *p* ≤ 0.05) between both groups. Grey dots are detected c-miRNAs that did not pass the thresholds for statistically significant expression (|Log2FC| ≤ 0.58 and *p* ≥ 0.05). (**e**). Corresponding heatmap visualizing the expression profiles of these differentially expressed c-miRNAs, where color gradients reflect deviations of each sample’s Log2FC from the mean Log2FC across all samples and dendrograms depicting sample statistical clustering. Color gradient indicates Log2FC values ranging from Log2FC = −2 (light green) to Log2FC = 2 (light red). (**f**). KEGG enrichment bar graph displaying 10 selected gene unions associated with the 4 differentially expressed c-miRNAs shown in (**e**). Statistical significance for volcano plots was determined using *t*-tests with raw *p*-values (*p* < 0.05). For the KEGG enrichment analyses, significantly enriched pathways were determined using FDR-adjusted *p*-values < 0.05 and are presented as −log10(*p*-value) (DIANA-miRPath v3.0).

Acknowledging the small pilot patient cohort used in this discovery experiment, differential expression analysis identified multiple c-miRNAs exceeding the background threshold in saliva and serum samples (Figure 2). Importantly, *p*-value adjustment using the Benjamini–Hochberg method of estimating false discovery rates (FDR) did not yield a *p*-adj ≤ 0.05 for any c-miRNAs from any biofluid, likely due to small sample size. Therefore, raw *p*-values were used to determine statistical significance. Based on this analysis, 55 c-miRNAs were detected in saliva (Figure 2a), of which 16 were significantly upregulated in BC patients (Figure 2b).

To predict the mechanistic roles of these c-miRNAs, Kyoto Encyclopedia of Genes and Genomes (KEGG) enrichment analysis was conducted using DIANA-miRPath v3.0 (gene union analysis), which aggregates predicted gene targets across multiple miRNAs. By assessing the 16 upregulated c-miRNAs within BC patient saliva, a significant enrichment in pathways associated with cancer, cell cycle, regulation of stem cell pluripotency, and estrogen signaling was found, suggesting that these miRNAs may be associated with BC (Figure 2c).

In serum, 37 c-miRNAs were identified (Figure 2d), with 4 significantly downregulated in the BC patient group (Figure 2e). Similar to the saliva analysis, KEGG enrichment analysis highlighted statistically significant enrichment of cancer-associated genes unions (e.g., viral carcinogenesis and p53 signaling pathway). However, broad biological functions (e.g., adherens junction, lysine degradation, and fatty acid metabolism) were also dominant, indicating that this enrichment profile may reflect generalized cellular processes rather than distinct disease-related pathways (Figure 2f).

Although 70 c-miRNAs exceeded the detection threshold in plasma, none showed significant expression differences between both groups, likely due to high intra-group variability, evidenced by poor intra-group data clustering (Figure 1a(iii)). As such, plasma data were excluded in further analyses. Supplementary Table S1 shows fold change, *p*-values, and adjusted *p*-values of these 70 detected c-miRNA within plasma. Given the large number of differentially expressed c-miRNAs in saliva, representing a less invasive collection method compared to blood-derived fluids, combined with the greater enrichment of cancer-related processes, saliva was selected as the focus for subsequent analyses and validation in a larger patient cohort.

### 2.3. Assessment of miRNAs Released by Canine and Equine Mammosphere-Derived Epithelial Cells (MDECs)

**Figure 3:**
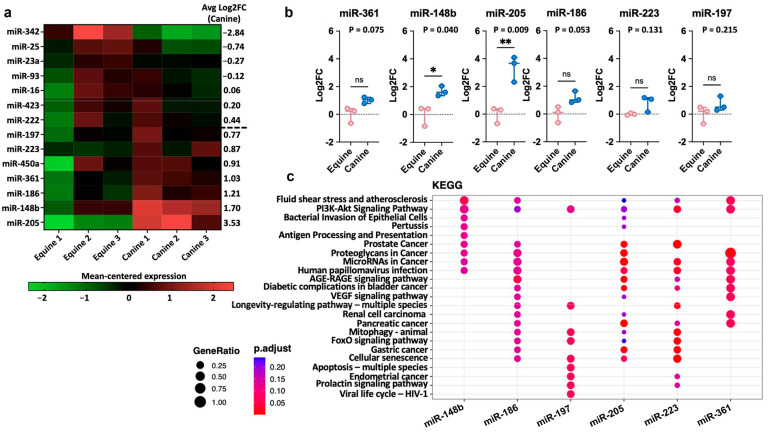
Six circulating microRNAs (c-miRNAs) detected at higher levels in the saliva of BC patients compared to controls, are also secreted at higher levels by canine (high incidence mammary cancer) mammosphere-derived epithelial cells (MDECs) when compared to equine (low incidence mammary cancer) MDECs. (**a**) Heatmap illustrating relative miRNA expression differences between canine and equine conditioned medium (CM). FC was calculated using the 2^−ΔΔCt^ method, then transformed via the Log2(FC) function. The miRNAs assessed in this comparative species approach were identified through the pilot Nanostring nCounter saliva analysis and are annotated in both the EquCab2.0 (equine) and CanFam3.1 (canine) genomes. Color intensity represents mean-centered expression of each sample’s Log2FC from the mean Log2FC across all samples (mean-centered expression = Log2FC of sample—Log2FC mean of all samples). Log2FC values are shown alongside the heatmap to indicate relative expression differences, with all expression levels normalized to a cel-miR-39 spike-in control. (**b**) Comparative plots showing miRNAs that were significantly differentially expressed between canine and equine MDEC-derived CM (i.e., miR-148b and miR-205) (*p* < 0.05) and those exhibiting a trending upregulation in canine MDECs (i.e., miR-361, miR-186, miR-223 and miR-197) (> Log2(1.5-fold), i.e., Log2FC = |0.58|), *p* > 0.05). Each point represents a different MDEC culture (collected from 3 individual animals) per species. Statistical significance was determined using an unpaired Welch’s *t*-test (* = *p* < 0.05, ** = *p* < 0.01, ns (not statistically significant) = *p* > 0.05 (**c**) Dot plot of KEGG functional enrichment analysis of validated miRNA target genes using MIENTURNET. Dot colors indicate Benjamini–Hochberg corrected *p*-adjusted values, and dot sizes indicate the ratio of genes targeted by the miRNA divided by the total genes within that pathway. The 23 KEGG pathways presented represent all pathways that achieved statistical significance (FDR-adjusted *p*-value < 0.05) following Benjamini–Hochberg correction across all KEGG pathways tested by MIENTURNET. No post hoc biological relevance filtering was applied.

Given that c-miRNAs are derived from a vast number of cell types throughout the body, we sought to define which saliva-derived c-miRNAs are: i) released by mammary cells and ii) released at greater concentrations by mammals at intrinsically greater risk of mammary malignancy. Mammosphere-derived epithelial cells (MDECs) are heterogeneous primary cell populations isolated from mammary tissue of healthy, non-pregnant mammals [29,30]. These cultures contain a mix of luminal and myoepithelial cell types and are particularly enriched for mammary stem and progenitor cells that are believed to be the origin of many BC subtypes [30,31]. In this study, we used MDECs obtained from dogs (canines) and horses (equines) to parallel human populations with or without early-stage BC that are inherently at a higher and lower risk of developing BC, respectively, to determine which c-miRNAs secreted by mammary cells might serve as potential biomarkers for BC. To refine the list of candidate c-miRNAs identified using Nanostring analysis of the human BC patient and control saliva samples (Figure 2b), we measured the expression of these miRNAs in canine and equine MDEC secretions, collected as conditioned media (CM), using reverse transcriptase (RT)-quantitative (q)PCR (Figure 3).

Among the 16 c-miRNAs found to be differentially expressed in saliva (Figure 2b), 14 had annotated orthologs in both dogs and horses. Two c-miRNAs were excluded from further analysis, namely miR-4454+7975, which is only annotated in humans, and miR-203a-3p, which is annotated in humans and dogs, but not in horses (https://mirbase.org/, accessed 14 November 2022).

All data were normalized to equine MDEC CM expression. Relative to expression levels in equine MDEC CM, five miRNAs (miR-23a, miR-93, miR-16, miR-423, and miR-222) exhibited comparable expression in both equine and canine MDEC CM, with Log_2_ fold changes (Log2FC) ranging between approximately −0.5 to 0.5 (Figure 3a). Two miRNAs, miR-342 and miR-25, were downregulated in canine MDEC CM when compared to equine MDEC CM (Log2FC ≤ −0.58), with miR-342 showing statistical significance (*p* = 0.026) whereas miR-25 did not (*p* = 0.629) (Figure 3a). Interestingly, seven miRNAs demonstrated elevated expression (Log2FC ≥ 0.58) in canine MDEC CM (Figure 3a), a pattern that aligned with the expression profiles observed in saliva from BC patients (Figure 2b). Within that list, two miRNAs were significantly upregulated (*p* < 0.05), namely miR-148b and miR-205 (Figure 3b). Based on the limited MDEC CM sample size (*n* = 3 per species), we decided to also select four additional miRNAs exhibiting a trend of upregulation, albeit not significant (*p*-values between 0.053 and 0.215), for further validation in the larger patient cohort, namely miR-361, miR-186, miR-223, and miR-197 (Figure 3b). In contrast, miR-450a was excluded for further validation due to inconsistent expression within the 3 equine MDEC CM samples (*p* = 0.4605; Figure 3a). To assess whether the six miRNAs identified through our filtering framework share target gene pathways, we performed enrichment analysis using the web-based tool MIENTURNET (Figure 3c). Statistical filtering identified 23 functional pathways composed of validated gene targets shared by at least two miRNA candidates. Notably, several cancer-related pathways (e.g., proteoglycans in cancer and microRNAs in cancer) were enriched, along with a pathway associated with prolactin signaling, a key regulatory axis in mammary gland development and function [32]. As these miRNAs were derived from non-malignant primary cell populations, their increased secretion may reflect underlying biological processes associated with breast tissue physiology and could indicate potential utility in predicting malignancy risk.

### 2.4. Study Population and Saliva Sample Analyses from a Larger Patient Cohort

Saliva samples from a larger validation cohort were analyzed using two different normalization controls. To refine the sample selection, we decided to exclude individuals with known pathogenic variants linked to breast cancer predisposition (e.g., BRCA1, BRCA2, PALB2) as well as patients who had undergone chemotherapy (e.g., taxol) before saliva collection, given that such treatments are known to alter c-miRNA expression patterns [33,34]. This resulted in saliva samples from 29 BC patients along with 14 age-matched control patients who had no prior history of cancer and were admitted to the hospital primarily due to female pelvic organ prolapse (Supplementary Figure S3). Detailed patient clinical data are provided in Supplementary Table S2 and breast tumor status of the BC patient group is shown in Table 2.

**Figure 4:**
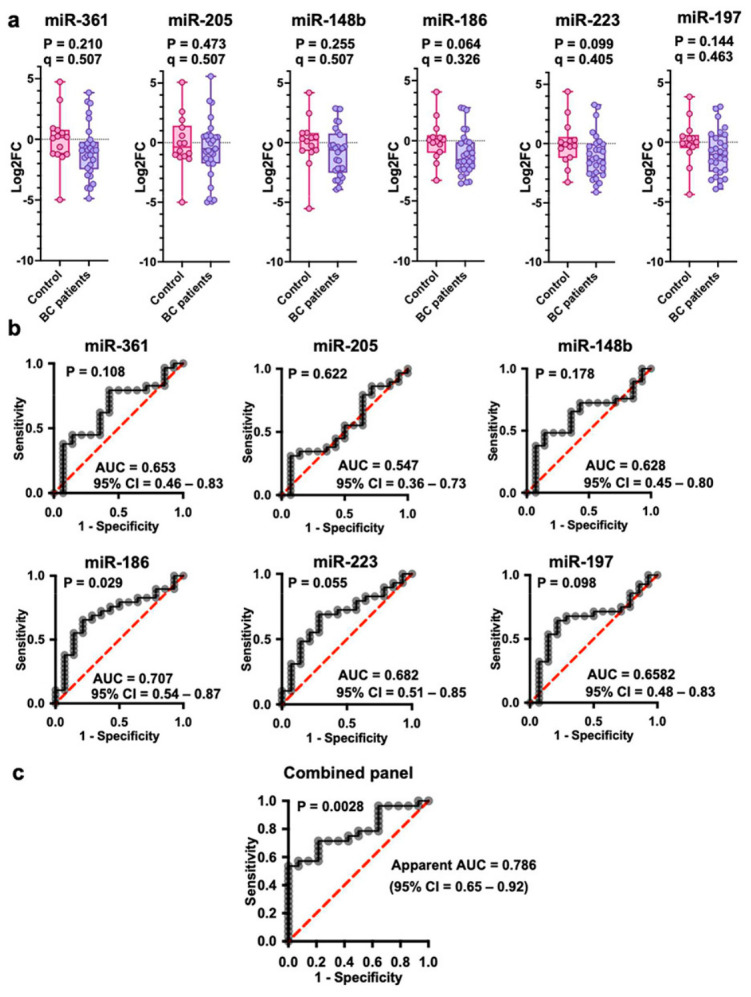
Candidate circulating microRNA (c-miRNA) expression in a validation cohort of control and BC patients normalized to endogenous RNU6 control. (**a**) RT-qPCR validation of selected c-miRNA candidates identified as differentially expressed between equine and canine MDEC-derived conditioned media (Figure 3). Expression levels were normalized to RNU6 and reported as Log2FC values, calculated using the 2^−ΔΔCt^ method. Each data point corresponds to an individual control or BC patient sample. Statistical significance was assessed using an unpaired Welch’s *t*-test (*p* < 0.05). Adjusted *p*-values (q-values) were performed using the Two-stage step-up (Benjamini, Krieger, and Yekutieli) method. (**b**) Receiver operating characteristic (ROC) analysis of the candidate c-miRNAs comparing control and BC patient groups. Each curve displays the corresponding *p*-value, area under the curve (AUC), and 95% CI, indicating the predictive capacity of each biomarker. *n* = 29 BC patients and *n* = 14 control patients. (**c**) Multivariate analysis of all six c-miRNAs. ROC obtained via multiple logistic regression analysis. Apparent AUC and 95% CI are shown.

Total RNA was isolated using a column-based approach based on spin column chromatography to mimic the RNA isolation used for the Nanostring nCounter pilot analyses described under 2.1. The c-miRNAs (miR-361, miR-205, miR-148b, miR-186, miR-223, miR-197) selected for analysis were based on their differential expression profiles observed in the MDEC CM experiments (Figure 3). Prior to statistical analysis, normal distribution of the data was validated by the Shapiro–Wilk test and plotted as quantile-quantile (QQ) plots (Supplementary Figure S4). No statistically significant differences in expression were observed between saliva samples from the control and BC groups, irrespective of whether normalization to cel-miR-39 spike-in (Supplementary Figure S5a) or to the U6 small nuclear-1 (RNU6) endogenous housekeeper [35,36] was used (Figure 4a).

To assess univariate predictive capabilities, receiver operating characteristic (ROC) analyses were performed [37] despite the lack of differences in mean expression. Univariate analyses for the majority of these miRNAs showed that these c-miRNAs were not discriminatory for control or BC, with area under the curve (AUC) values ranging between 0.5 and 0.7 (Figure 4b), indicating inadequate predictive capabilities for individual c-miRNAs. Although the predictive capacity of miR-186 was statistically significant and showed an AUC > 0.7 (AUC = 0.707), the 95% confidence interval (CI) of 0.54–0.87 demonstrated weak predictive sensitivity (Figure 4b). Quantification of the same selected c-miRNAs, again, did not yield statistically significant differences between the two groups when cel-miR-39 spike-in normalization was used (Supplementary Figure S5a) and AUC values ranged between 0.5 and 0.6, indicative of inadequate predictive capabilities for individual c-miRNAs (Supplementary Figure S5b).

While individual c-miRNA candidate levels displayed weak predictive capacity, multivariate analysis revealed that their combined expression pattern modestly enhanced apparent discriminatory power (*p* = 0.0028, apparent AUC = 0.786, 95% CI 0.65–0.92) (Figure 4c). However, given that this model was both trained and evaluated on the same dataset, we subsequently utilized two internal cross-validation approaches, namely the repeated 5-fold cross validation AUC and leave-one-out cross validation (LOOCV) AUC, to more reliably assess predictive performance (Table 3). Upon cross-validation, the predictive capacity was substantially mitigated, with a 5-fold AUC = 0.616 and LOOCV AUC = 0.599, indicating that this panel is poorly predictive. Notably, the predictive capacity of the panel when normalized to cel-mir-39 was further mitigated (Supplementary Figure S5c, Table 3), indicating that alternative normalization strategies or an optimized miRNA panel may enhance predictive capacity.

To further optimize this multivariate predictive panel, miRNAs that displayed an apparent AUC > 0.65 (i.e., miR-361, miR-186, miR-223, miR-197) (Figure 4b) were again analyzed using multiple logistic regression to determine if combinatorial expression patterns resulted in a further enhanced capacity (Supplementary Figure S6). While statistically significant (*p* = 0.029) with an apparent AUC > 0.7, the 95% CI between 0.55 and 0.86 indicates weak predictive specificity. Internal cross validation revealed AUC values that indicates that this alternative panel is not predictive (5-fold AUC = 0.547, LOOCV AUC = 0.510) (Table 3). Importantly, the predictive capability of this panel was roughly equivalent to that of the miR-186 univariate analysis, further indicating the combined analysis of the best performing predictive miRNAs does not sufficiently produce a predictive c-miRNA panel. Still, further analysis of these c-miRNAs is warranted, as RNU6 has been reported to be upregulated in tumor tissues and biofluids of BC patients [38,39,40]. Importantly, although statistically non-significant, these data suggest that the combined c-miRNA panel may have some potential in predicting BC presence in human patients, particularly given that normalization strategy appeared to be a crucial aspect of enhancing predictive capacity.

## 3. Discussion

The present study demonstrates how a comparative species framework can inform the discovery of circulating microRNAs (c-miRNAs) that signal mammary cancer susceptibility across species with contrasting cancer incidence (Supplementary Figure S7). Dogs, which display high rates of spontaneous mammary neoplasia, and horses, which rarely develop such tumors, represent natural extremes of mammary cancer risk [20,21,22,23,24,25]. While such cross-species model can provide insights that may not be observed when focusing on only one single species, there are inherent limits to comparing cellular mechanisms, in this particular study c-miRNAs, between dogs and horses. For example, the reproductive biology and endocrine environment of dogs and horses differ [41,42] and domestic dogs typically live between 6 and 15 years depending on breed [43], whereas it is common for horses to live for more than 20 years [44]. Additionally, mammary tumor incidence between dogs and horses may not directly reflect epithelial cell transformation in each species [45]. In this study, profiles of miRNAs secreted by in vitro cultures of mammosphere-derived epithelial cells (MDECs) isolated from dogs and horses were assessed. While evaluating miRNAs from MDECs ensured the cell types of origin, it cannot be automatically expected that cultured cells behave exactly as they do in vivo [46]. Despite these limitations, integrating mammary cell data from canine and equine models with salivary profiles from human subjects, allowed for the identification of conserved candidate miRNAs that may mark baseline predisposition for malignant transformation.

Moreover, such comparative oncology perspective might add evolutionary and mechanistic depth to biomarker identification. Previous work has shown that mammary stem and progenitor cells retain conserved signaling hierarchies across species and that disruptions in these regulatory circuits contribute to tumorigenic potential [29,30,31]. In the present context, identifying c-miRNAs that align with species-specific risk patterns provides an additional filter for functional relevance. This framework enhances interpretability by connecting differential expression of c-miRNAs in biofluids with conserved mammary cell biology rather than relying solely on statistical enrichment in human cohorts.

While the comparative approach strengthens biological inference, the modest cohort size used in this study limited statistical power and constrained the generalizability of the findings. Some c-miRNA candidates exhibited expression trends are consistent with previously reported breast cancer (BC)–associated miRNAs; however, their expression did not reach statistical significance [12,14,18]. A valuable approach to improve statistical rigor would be to re-examine these near-significant miRNAs in expanded populations to evaluate their reproducibility and potential additive effects when analyzed in combination. Given that this current work focused on luminal breast tumor subtypes, further expanding the population cohort to include triple negative breast cancer subtypes, which are common in canine [47,48], would improve the generalizability of this predictive framework. Importantly, and although such samples are currently unavailable to us, future efforts should include longitudinal sampling, where individuals are monitored over time to assess whether specific c-miRNA shifts precede overt pathology, before any conclusions can be made on useful and validated indicators of BC risk. Such within-subject comparisons are critical for early detection studies, as they control for genetic and environmental heterogeneity and provide temporal resolution to evaluate predictive validity [6,28]. Vice versa, our control group consisted of individuals who were primarily admitted to SUNY Upstate Medical University for pelvic organ prolapses and thus, do not represent true “healthy” patients. While none of these control patients had a history of any type of cancer, they were not pre-screened for genetic risk factors and we cannot exclude any potential co-founding factors influencing the c-miRNA profile of these individuals. Specifically, miR-222, which was highlighted by our Nanostring nCounter pilot analysis of patient saliva, has always been shown to be elevated locally in the uterosacral ligaments of patients with pelvic organ prolapse [49]. One should ideally include a truly healthy control population to extrapolate these findings to real-world screening settings.

Although saliva was the focus of this present study, additional biofluids warrant examination to define the context in which particular miRNAs are most informative. Saliva is uniquely well suited for non-invasive screening because it can be collected repeatedly without specialized training and reflects systemic physiology through exosome-associated cargo derived from multiple tissues [14,15,16,17]. However, several studies have demonstrated that c-miRNA profiles vary substantially across fluids, with certain tumor-derived signatures being enriched in plasma or serum rather than saliva [50,51]. Multi-fluid comparisons, including plasma, serum, and even urine, could therefore improve the robustness of future diagnostic models by capturing complementary aspects of systemic and local signaling.

The relevance of saliva as a diagnostic medium extends beyond accessibility. Salivary miRNAs have shown potential as biomarkers in breast, oral, pancreatic, and other malignancies, often mirroring molecular changes occurring at distant tumor sites [14,15,16,17,52]. These observations highlight saliva’s capacity to reveal early tumorigenic signals before morphological abnormalities become detectable by imaging. Nonetheless, biofluid-specific variability remains a challenge, and standardized collection, storage, and normalization protocols are essential to ensure reproducibility across studies [53,54,55]. Differences in diet, circadian rhythm, and oral inflammation can alter salivary miRNA composition, reinforcing the need for internal reference controls with stable expression across conditions [38,51].

The predictive strength of individual miRNAs is often limited, and most clinically promising assays employ multiplexed panels that capture the combined effect of several dysregulated transcripts. For instance, combinations of c-miRNAs have achieved diagnostic accuracies exceeding 85% in early-stage breast cancer and other epithelial malignancies [56,57]. Many of these panels include recurrent candidates such as miR-21, miR-155, and miR-200c, which have been implicated in tumor growth, immune modulation, and epithelial-to-mesenchymal transition [14,15]. In this current study, multivariate analysis of the combined c-miRNA panel (miR-361, miR-205, miR-148b, miR-186, miR-223, and miR-197), regardless of normalization to internal RNU6 or spike-in cel-miR-39, did not yield predictive capacity suitable for BC detection appropriate for clinical diagnosis. While absent of robust predictive capacity, these data demonstrate a novel method of refining predictive biomarkers, and expanding this analysis to a larger cohort of more pathologically diverse patients may significantly improve the statistical rigor. Such multi-analyte strategies are likely to represent the most practical path toward clinical implementation. Incorporating conserved candidates identified through a comparative framework could refine such panels by enriching for biologically relevant features rather than purely empirical markers.

Beyond immediate diagnostic applications, c-miRNA signatures have the potential to improve personalized screening by identifying individuals at elevated risk who may benefit from intensified surveillance. Several studies have shown that total c-miRNA levels correlate with tumor burden and disease progression, suggesting that dynamic monitoring could provide an early signal of malignant transformation [56,57,58]. Translating this approach to saliva could extend these advantages to a fully non-invasive format suitable for repeated testing in community or home-based settings. The integration of longitudinal design, multi-fluid analysis, and cross-species validation may therefore accelerate the development of next-generation screening tools capable of detecting BC risk with high precision and minimal patient burden.

This work supports the feasibility of combining comparative oncology with non-invasive molecular profiling to uncover conserved miRNAs linked to BC detection and susceptibility. Despite the limited sample size and statistical non-significance, the results highlight candidates with potential translational value and underscore the importance of extending validation through longitudinal, multi-cohort, and multi-fluid analyses. The continued refinement of miRNA-based panels and standardization of analytic protocols will be essential to realize their full potential for early BC detection and personalized risk assessment.

## 4. Materials and Methods

### 4.1. Patient Information

Coded patient biofluid samples were obtained from the State University of New York (SUNY) Upstate Medical University Biorepository Core in Syracuse, NY (IRB no. 387215 approved by the SUNY Upstate Medical University IRB). Breast cancer (BC) in BC patients was confirmed through biopsy and histological evaluation, and patients without a history of BC (control patients) underwent surgery for reasons unrelated to cancer, such as pelvic organ prolapse. Age-matched patients bearing low-grade, luminal-like breast tumors with no prior history of cancer or detectable genetic predisposition to cancer were selected for this study. The specimens evaluated in this study were collected from patients who had not received chemotherapy or radiation treatment prior to biofluid collection. All samples were obtained from fasting patients immediately before surgery. Patient medical histories and clinical records were gathered by the Biorepository Core at SUNY Upstate Medical University and coded data was provided to the research team.

### 4.2. Patient Biofluid Collection

Participants who consented to provide unstimulated whole saliva samples for the SUNY Upstate Medical University Biorepository Core were instructed to spit into a sterile container prior to performing a routine mouthwash. Collected saliva samples were immediately chilled on ice, transferred to cryogenic vials, and subsequently stored at −80 °C. For serum collection, whole blood was drawn into sterile Vacutainer Serum Tubes (BD, Franklin Lakes, NJ, USA), allowed to clot for 30 min at room temperature (RT), and then centrifuged at 1250× *g* for 10 min at 4 °C. The serum fraction was separated, aliquoted into individual freezer tubes, flash-frozen in liquid nitrogen, and stored at −80 °C. For plasma collection, whole blood was collected in sterile Vacutainer EDTA tubes (BD, Franklin Lakes, NJ, USA), centrifuged under the same conditions (1250× *g* for 10 min at 4 °C), and the plasma layer was similarly aliquoted, frozen in liquid nitrogen, and stored at −80 °C. Prior to miRNA extraction, all samples were thawed on ice and processed immediately. All frozen samples underwent a single thaw immediately prior to analysis to preserve miRNA integrity.

### 4.3. Cell Culture of Mammosphere-Derived Epithelial Cells

Equine and canine mammary gland tissues were collected from research animals at Cornell University College of Veterinary Medicine (CVM) after euthanasia for reasons unrelated to this research. Mammary gland tissues (*n* = 3 per species) were obtained from clinically healthy, non-lactating adult females, immediately immersed in sterile phosphate-buffered saline (PBS), kept on ice, and processed within 24 h. Mammary gland tissues were processed into mammosphere-derived epithelial cells (MDECs) using established protocols, as previously described [59], and cultured in epithelial stem cell (EpSC) medium. EpSC medium consisted of a 1:1 mixture of DMEM/Ham’s F12, supplemented with 10% fetal bovine serum (FBS), 2% B27, 1% penicillin/streptomycin (all from Invitrogen, Carlsbad, CA, USA), and growth factors including 10 ng/mL basic fibroblast growth factor (FGF) and 10 ng/mL epidermal growth factor (EGF) (both from Sigma-Aldrich, St. Louis, MO, USA). Cells were maintained at 37 °C with 5% CO_2_ [30].

### 4.4. Generation of Conditioned Medium

Conditioned medium (CM) for cell-free microRNA (miRNA) analysis was collected following previously established protocols [60,61]. Low passage (*p* < 8) equine and canine MDECs were seeded at a density of 1 × 10^6^ cells in a T25 flask containing 8 mL of EpSC medium. After 24 h of incubation, EpSC medium was removed, and the cell monolayers were washed three times with PBS. Immediately after the final washing step, 8 mL of serum-free DMEM was added to promote miRNA secretion, and the cells were incubated for an additional 48 h. Following this incubation period, the cell culture supernatant was collected into sterile 15 mL tubes and centrifuged twice at 300× *g* for 10 min each to eliminate residual cell debris. Aliquots of 250 μL of the resulting CM were snap frozen in liquid nitrogen and stored at −80 °C until further analysis.

### 4.5. Nanostring nCounter

Total RNA for Nanostring nCounter (Nanostring Technologies, Seattle, WA, USA) analysis was extracted using the Midi Plasma/Serum RNA Purification Kit (Norgen Biotek, Thorold, ON, Canada). Serum, plasma, and saliva samples were thawed on ice. To remove cell debris, saliva was centrifuged at 10,000× *g* for 10 min, while serum and plasma samples were spun at 400× *g* for 3 min. One mL of supernatant was collected from each sample and processed according to the manufacturer’s instructions, with RNA eluted at 100 µL volumes across all treatment groups.

Following extraction, RNA samples were concentrated and purified using linear acrylamide. Each 100 μL RNA sample received 300 μL of ice cold 100% EtOH, 10 μL 3M sodium acetate (pH 5.5) (Invitrogen), 3 μL linear acrylamide (5mg/mL) (Invitrogen), and were incubated at −80 °C for 16 h. Samples were centrifuged at 13,000× *g* for 30 min at 4 °C to pellet the RNA, followed by two washes with 75% ethanol. After air drying, RNA pellets were responded in 10 μL of nuclease-free water.

RNA concentration and purity were assessed using a NanoDrop 2000 spectrophotometer (Thermo Fisher Scientific, Waltham, MA, USA). Due to low RNA yield and purity from biofluids [62], samples with 260/280 ratios between 1.5 and 1.8 were deemed acceptable for Nanostring analysis based on manufacturer’s consultation. RNA samples were diluted to 33.33 ng/μL (3 µL total, equivalent to 100 ng RNA) and submitted to the NYU Langone Health Division of Advanced Research Technologies (DART) for analysis with the Human v3b miRNA CodeSet Kit (Nanostring Technologies). Normalization of code-set data was performed using linear standard curves from Positive Controls (Pos A-F) and Ligation Positive Control (A and B) via nSolver Analysis Software v4.0 (Nanostring Technologies). miRNAs with maximum normalized counts below 50 were excluded to minimize background noise [28], following manufacturer’s recommendations. Additionally, miR-451a was removed from the analysis due to its known association with hemolysis contamination [63].

Finally, ligation-normalized datasets were visualized, and statistical analyses (*p*-values, fold changes) were conducted using the ROSALIND^®^ platform (www.rosalind.bio; ROSALIND^®^, San Diego, CA, USA; accessed on 21 November 2022) [64,65,66,67,68]. Fold changes, Log2FC, raw *p*-values, and significance were calculated using *t*-tests (See Section 4.8).

### 4.6. miRNA Pathway and Functional Enrichment Analysis

Pathway enrichment analysis was performed using DIANA-miRPath v3.0 (http://www.microrna.gr/miRPathv3, accessed on 18 April 2026). Regarding background gene set, experimentally validated human miRNA–target interactions derived from DIANA-TarBase v7 was utilized. Kyoto Encyclopedia of Genes and Genomes (KEGG) pathway enrichment was evaluated using Fisher’s exact test, with Benjamini–Hochberg correction. Analysis was conducted using the union of target genes across all input miRNAs to assess the collective pathway-level effects of miRNA panels obtained from either saliva (saliva-derived panel; *n* = 16) or serum (serum-derived panel; *n* = 4) samples. Significantly enriched pathways were defined as those with FDR-adjusted *p*-values < 0.05 and are presented as −log10 (*p*-value). From among statistically significant pathways, those biologically relevant to the study context were prioritized for presentation, specifically pathways associated with cancer signaling, stem cell regulation, hormonal signaling, and cell proliferation and growth. This biological filtering was applied to focus interpretation on pathways mechanistically relevant to breast cancer pathogenesis, consistent with the study’s diagnostic objectives.

Functional enrichment analysis of miRNA target genes was performed using MIENTURNET (R version 4.3.1) (http://userver.bio.uniroma1.it/apps/mienturnet/, accessed on 18 April 2026), a web-based tool for miRNA-target enrichment and network analysis [69]. A subset of six miRNAs, provided as mature miRBase identifiers for *Homo sapiens*, was selected for analysis with miRbase ID selected as the data input type. Experimentally validated miRNA-target interactions were retrieved using the miRTarBase database (version 10.0) within MIENTURNET. Pathway enrichment analysis was conducted against the KEGG database. The background gene set comprised all experimentally validated target genes present in miRTarBase (version 10.0), which represents the default reference universe in MIENTURNET, as the tool does not support user-defined background gene sets.

Enrichment significance was assessed using statistical methods implemented in MIENTURNET, with multiple testing correction applied to control for false discovery. Enriched pathways were visualized using dot plots, with significance represented as adjusted *p*-values. Resulting plots were exported for figure generation.

### 4.7. RNA Extraction and Real-Time Quantitative PCR (RT-qPCR)

For pilot and large cohort analyses, total RNA was isolated from biofluids and CM using the Total RNA Purification Kit (Norgen Biotek, Thorold, ON, Canada), following protocols optimized for serum/plasma samples. In brief, 350 μL sample was transferred to 1.5 mL microcentrifuge tubes. Saliva samples were centrifuged at 10,000× *g* for 10 min to remove debris, while serum, plasma, and CM were centrifuged at 300× *g* for 4 min. From each sample, 200 μL supernatant was combined with 600 μL of Buffer RL (Norgen Biotek) supplemented with 1% β-mercaptoethanol (BME).

Immediately after lysis buffer addition, human biofluid samples received 3 μL of 33.33 fm/μL cel-miR-39 spike-in (cel-miR-39 mirVana miRNA mimic, Ambion, Austin, TX, USA) (total of 100 fm). CM samples received 3 μL of 6.66 fm/μL cel-miR-39 spike-in (total of 50 fm). After vortexing, 800 μL of 100% ethanol was added, and RNA isolation was completed following the manufacturer’s protocol. All RNA was eluted in 55 μL nuclease-free water. Due to difficulties in quantifying RNA concentrations from liquid biopsy samples via spectrophotometric (Nanodrop) (Thermo Fisher Scientific, Waltham, MA, USA) and fluorometric (Qubit) (Thermo Fisher Scientific) methods, fixed volumes were used for downstream reverse transcription (RT) reactions and RT-qCR rather than concentration-based quantifications. All miRNA expression was normalized to cel-miR-39 spike-in control to account for technical variability [70,71,72,73], or RNU6 to account for biological variability [35,36].

All cDNA synthesis was performed using the Taqman MicroRNA Reverse Transcription Kit (Thermo Fisher Scientific), and miRNA expression levels were assessed using Taqman MicroRNA Assays (Life Technologies, Carlsbad, CA, USA). For expression normalization, cel-miR-39 was used as a reference control for canine and equine CM, and human biofluids. Comparative expression analyses were conducted by normalizing BC patient samples to controls, and canine CM samples to equine CM samples, using the 2^−ΔΔCt^ method (reported as fold change). U6 small nuclear-1 (RNU6) was also evaluated as an endogenous housekeeper to assess differences in normalization when compared to the spike-in. Supplementary Table S3 contains a list of all miRNA primers for RT-PCR, including the TaqMan MicroRNA Assay IDs.

Heatmaps for RT-qPCR data were generated by transforming fold change values into log_2_ scale. To facilitate visualization of relative expression patterns across samples, log2FC values were mean-centered for each miRNA by subtracting the cross-sample mean log2FC, such that the plotted values represent the deviation of each sample from the mean expression across the cohort (Mean-centered expression = Log2FC of sample − Log2FC mean of all samples). For larger cohort comparisons, FC values were converted into Log_2_FC directly due to high sample-to-sample variation.

### 4.8. Statistical Analysis

All data processing and visualization was conducted through the ROSALIND^®^ platform (ROSALIND^®^, San Diego, CA, USA). Read Distribution violin plots, identity heatmaps, and sample multidimensional scaling (MDS) plots were generated during quality control steps.

Ligation control-normalized Nanostring nCounter miRNA counts were uploaded to the ROSALIND ^®^ platform for analysis and data visualization. Fold changes, Log2FC values, *p*-values, and optional covariate corrections were calculated using the *limma* R package. Clustering of differentially expressed miRNAs was performed using the Partitioning Around Medoids (PAM) method via the fpc R package [61,62,63,64,65]. R analysis performed in RStudio (version 2024.12.1+563). Subsequently for visualization, these miRNAs were displayed as a heatmap in which sample relationships and within-cluster feature ordering were determined by hierarchical clustering, shown as dendrograms. For determining statistically significantly differentially expressed c-miRNAs for follow-up analysis with MDEC CM, c-miRNAs with Log2FC ≤ −1.5-fold change or ≥ 1.5-fold change (Log2FC = |0.58|) and a *p*-value < 0.05 were selected.

Welch’s *t*-tests were performed using GraphPad Prism 10 (version 10.3.0) (GraphPad, La Jolla, CA, USA). Receiver operating characteristic curve (ROC) analyses were also carried out in Prism to evaluate the diagnostic potential of individual miRNAs [73]. Adjusted *p*-values (q-values) were obtained following Welch’s *t*-tests corrected using the Two-stage step-up (Benjamini, Krieger, and Yekutieli) method. Multivariable logistic regression was performed to assess the combined diagnostic utility of multiple miRNAs. Cancer status (1 = cancer, 0 = control) was used as the dependent variable, and miRNA expression levels (Log2FC) were included as predictors. Model-derived probabilities were used to construct ROCs, and the area under the curve (AUC) was calculated with 95% confidence intervals to evaluate discrimination. AUC significance was tested against the null hypothesis of 0.5. Model calibration was assessed using the Hosmer–Lemeshow test, and Tjur’s R^2^ was used to estimate explained variance. A probability threshold of 0.5 was used to generate classification metrics.

Out-of-sample discrimination was estimated by internal cross-validation using two complementary schemes: leave-one-out cross-validation (LOOCV) and repeated stratified 5-fold cross-validation (200 repetitions). For LOOCV, the predicted probabilities from all iterations were pooled and a single AUC was computed. For repeated 5-fold cross-validation, folds were stratified by outcome to preserve the case-to-control ratio. Out-of-fold predicted probabilities were pooled within each repetition to yield one AUC per repetition, and the 200 AUCs were summarized as the mean with the 2.5–97.5th percentile range. Optimism was quantified as the difference between the apparent and leave-one-out cross-validated AUC. Cross validation analysis was performed in R version 4.5.0 using the pROC package build under R version 4.5.1.

## Figures and Tables

**Table 1:** Patient information for saliva, serum, and plasma pilot analysis via Nanostring nCounter.

Patient (Pathotype & ID)	Age	Tumor Grade (1–4)	Estrogen Receptor (ER)	Progesterone Receptor (PR)	Human Epidermal Growth Factor Receptor (HER2)	Pathology Description
BC patient 1	62	1	+	+	-	Invasive carcinoma of no special type (NST)
BC patient 2	56	1	+	+	-	Invasive, carcinoma, cribriform
BC patient 3	52	1	+	+	-	Invasive, carcinoma, cribriform
Control 1	62	/	/	/	/	Uterine-related pathology. No history of cancer
Control 2	51	/	/	/	/	Uterine-related pathology. No history of cancer
Control 3	56	/	/	/	/	Benign urothelial issues. No history of cancer

**Table 2:** Breast tumor status of the larger patient cohort.

Receptor Expression Status								
ER ^1^	*n*	%	PR ^2^	*n*	%	HER2 ^3^	*n*	%
Positive	29	100	Positive	27	93.10	Positive	5	17.24
Negative	0	0	Negative	2	6.90	Negative	22	75.86
NA *	0	0	NA *	0	0	NA *	2	6.90
Tumor grade								
	*n*	%						
Grade 1	12	41.38						
Grade 2	13	44.94						
Grade 3	2	6.90						
Grade 4	0	0.00						
NA *	2	6.40						

^1^ Estrogen receptor (ER), ^2^ Progesterone receptor (PR), ^3^ Human epidermal growth factor receptor 2 (HER2), * Tumor information not available.

**Table 3:** Apparent and internal cross-validated predictive capacities of multivariate miRNA panels.

miRNA Panel	Apparent AUC	5-Fold AUC (200 Splits)	Leave-One-Out CV (LOOCV)	Optimism (Apparent—LOOCV)
RNU6 normalized	0.786 (95% CI = 0.65–0.92)	0.616 (95% CI = 0.53–0.70)	0.599	0.19
cel-miR-39 normalized	0.699 (95% CI = 0.54–0.86)	0.439 (95% CI = 0.33–0.55)	0.388	0.31
Refined RNU6 (apparent AUC > 0.65) normalized	0.709 (95% CI = 0.55–0.86)	0.547 (95% CI = 0.449–0.620)	0.510	0.20

## Data Availability

The Nanostring data presented in this study are openly available in NCBI GEO, number GSE319390.

## Supplementary Materials

The following supporting information can be downloaded at: https://www.mdpi.com/article/10.3390/ijms27146198/s1.

## Abbreviations

The following abbreviations are used in this manuscript:

AUC	Area under the curve
BC	Breast Cancer
BME	β-mercaptoethanol
BOADICEA	Breast and Ovarian Analysis of Disease Incidence and Carrier
BRCA1/2	Breast Cancer Associated Gene 1/2
cDNA	Complementary DNA
c-miRNA	Circulating microRNA
CM	Conditioned medium
CVM	College of Veterinary Medicine (Cornell)
DART	Division of Advanced Research Technologies (NYU Langone)
DMEM	Dulbecco’s Modified Eagle Medium
EDTA	ethylenediaminetetraacetic acid
EGF	Epidermal Growth Factor
EpSC	epithelial stem cell
ER	Estrogen Receptor
FC	Fold change
FGF	Fibroblast Growth Factor
GLOBOCAN	Global Cancer Observatory
HER2	Human epidermal growth factor receptor 2
HR	Hormone Receptor
MDEC	Mammosphere-derived epithelial cells
MDS	Multidimensional scaling
miRNA	MicroRNA
MRI	Magnetic Resonance Imaging
NST	No special type
PBS	Phosphate-buffered saline
PAM	Partitioning Around Medoids
PCR	Polymerase chain reaction
PLG	Phase lock gel
PR	Progesterone receptor
QC	Quality control
qPCR	Quantitative polymerase chain reaction
ROC	Receiver operating characteristic
RNU6	U6 small nuclear RNA
RNA	Ribonucleic acid
SUNY	State University of New York
