# Supplementary material for: A Comparative Species Framework to Identify Candidate Salivary miRNAs Associated with Breast Cancer Risk
Source: Int J Mol Sci. 2026 Jul 11;27(14):6198. doi: 10.3390/ijms27146198 (PMC13410475; doi:10.3390/ijms27146198)
Supplement: Supplementary file 1 [file ijms-27-06198-s001.zip › ijms-4310232-supplementary.pdf]

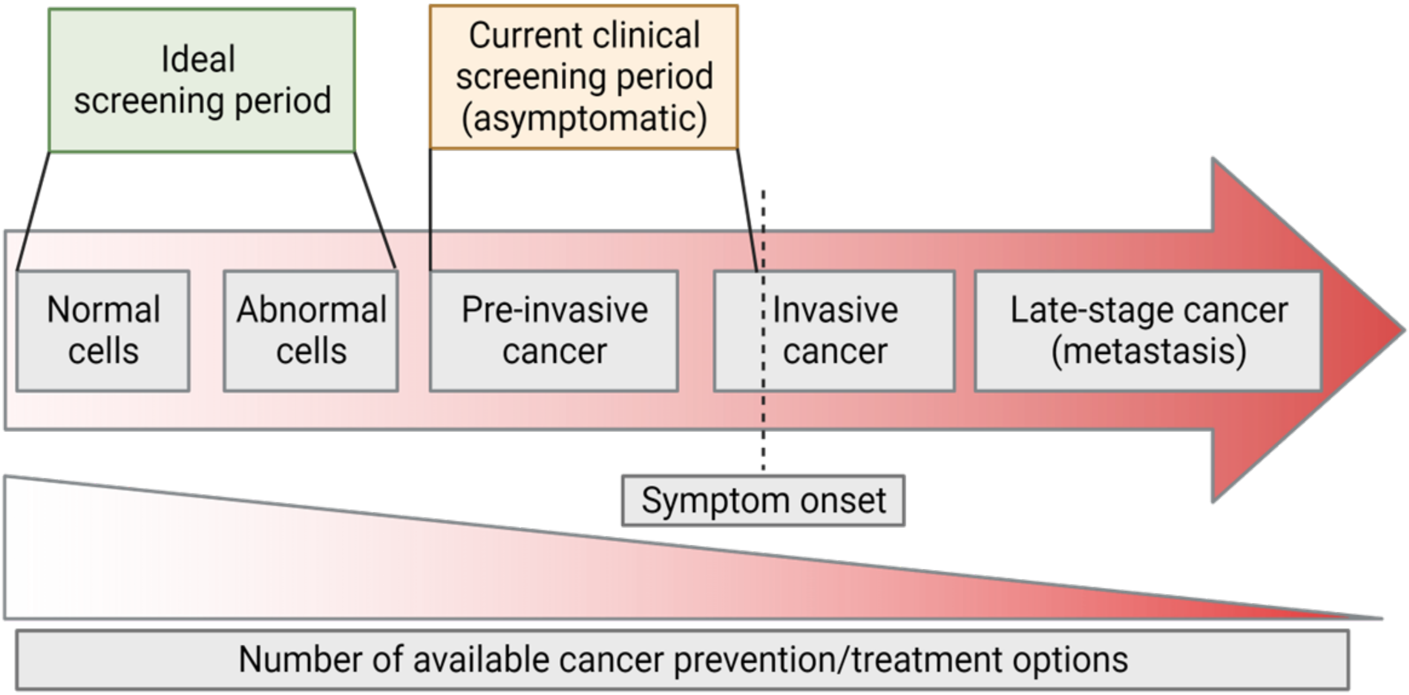

Supplementary Figure S1. Timeline of breast cancer (BC) screenings and opportunities for improvement. The diagram illustrates the current screening periods for BC (yellow), as well as the ideal period (green). Early screening before malignant transformation or symptom onset expands preventative and therapeutic options for patients. Adapted from [4].

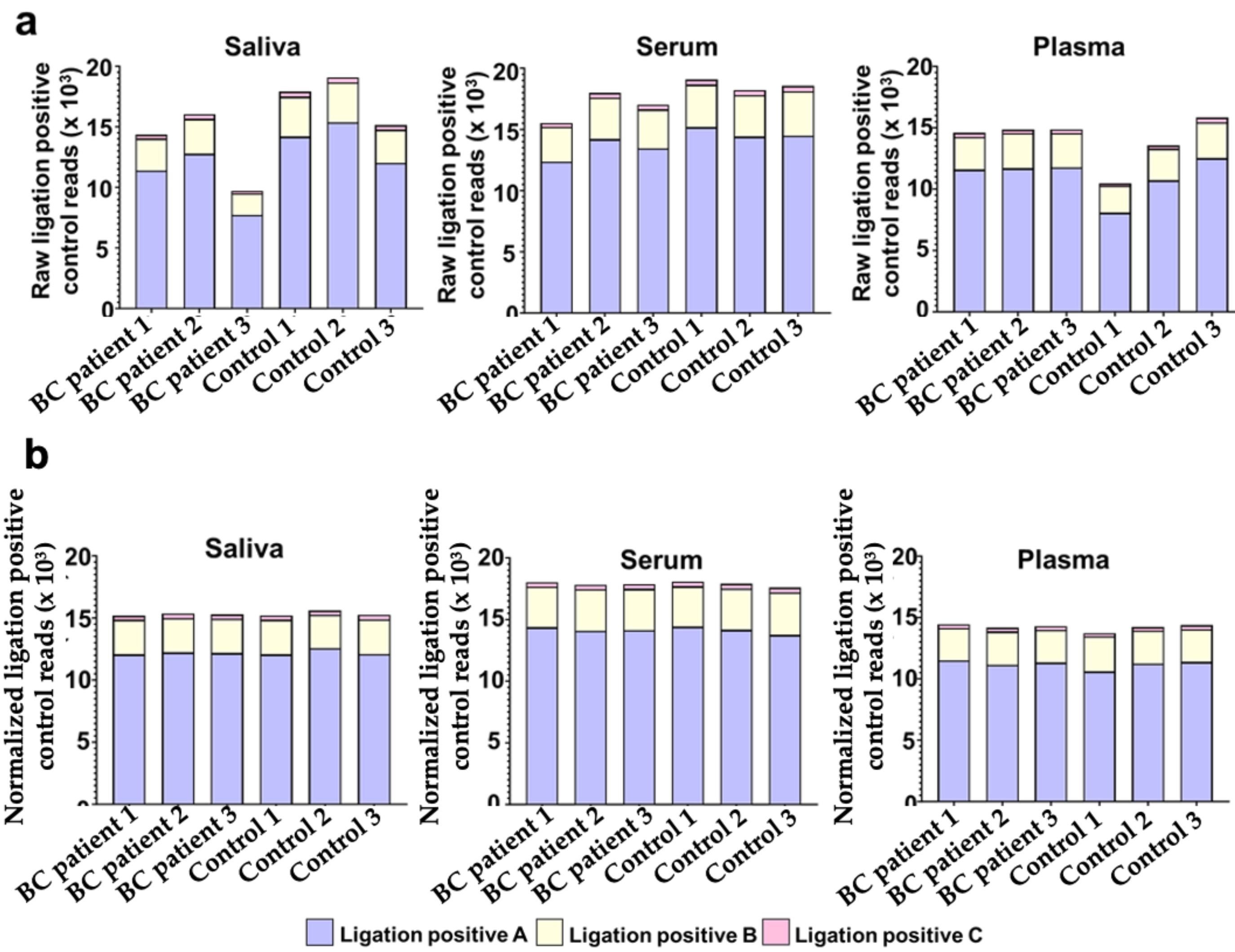

Supplementary Figure S2. Variations in ligation efficiency in NanoString nCounter analysis were addressed by normalizing reads to ligation positive controls. (a). Raw ligation control read counts for saliva, serum, and plasma. **(b).** Read counts after normalization for the same biofluids. Sample size: n = 3 per group (BC patient and Control).

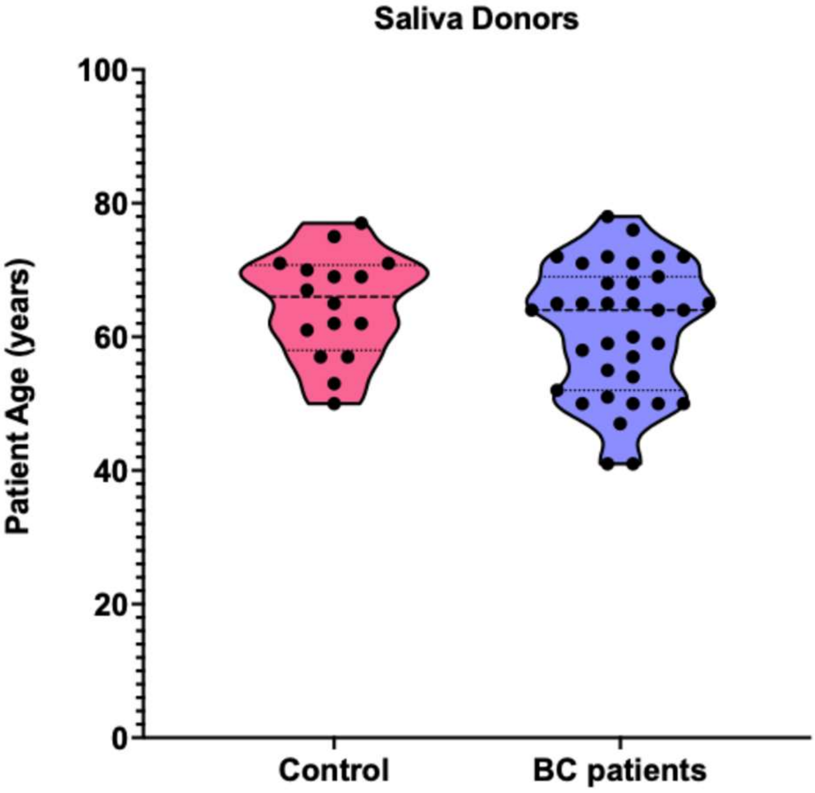

Supplementary Figure S3. Ages of control and BC patient saliva donors included in the larger cohort analysis. Violin plots of ages (in years) of control and BC patients. Data points represent individual patients. Central dotted line indicates median age for the study groups and upper and lower dotted lines indicate upper (Q3) and lower (Q1) quartiles. n=14 control and n= 29 BC patients.

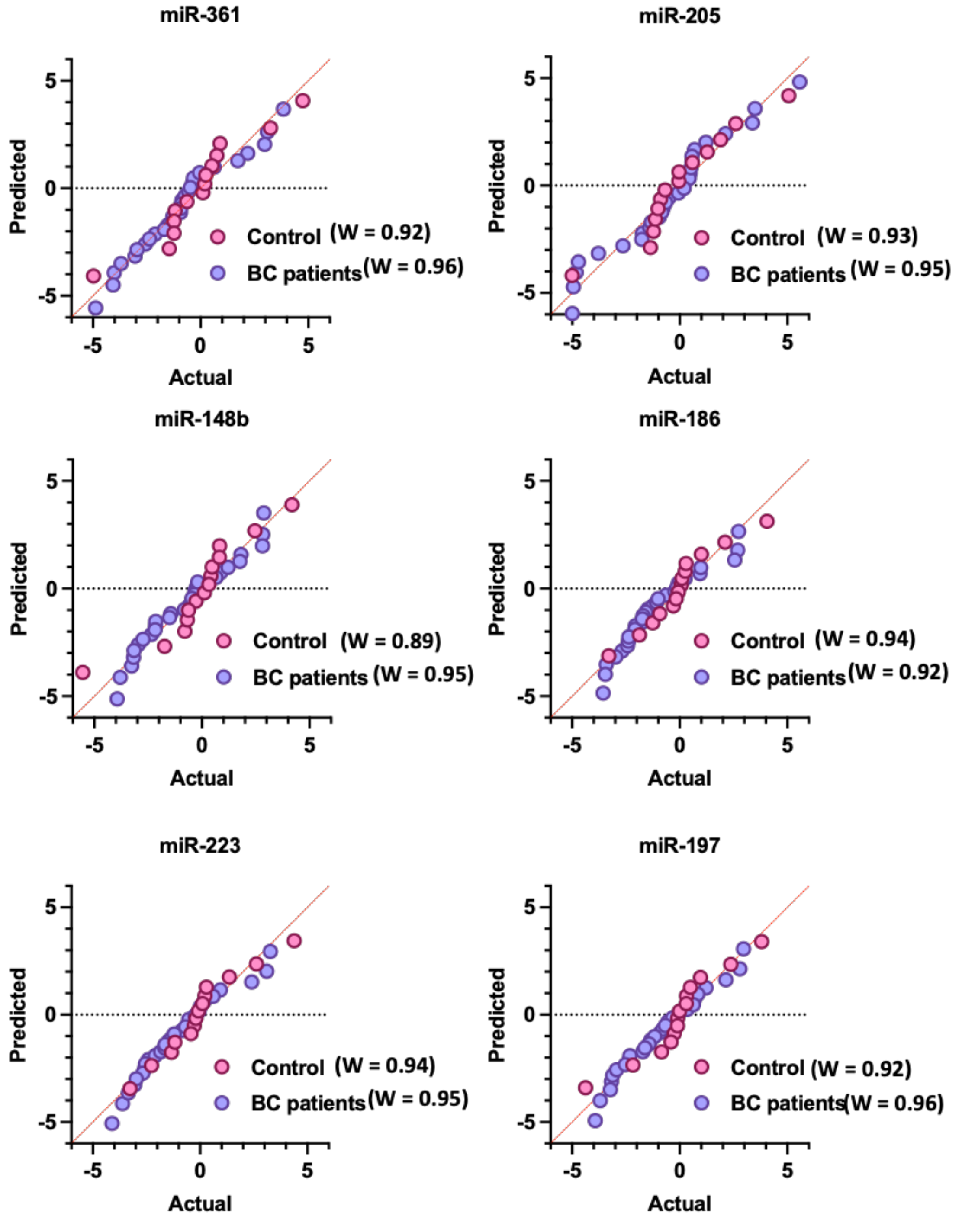

Supplementary Figure S4. Normal QQ Plots to validate normality of human saliva qPCR data. Quantile-quantile (Q–Q) plots assessing the distribution of Log2FC values for each miRNA in control and BC patient groups. The data for each miRNA generally follow a normal distribution, as indicated by alignment along the reference line. Normality was assessed using the Shapiro–Wilk test, with W-values approaching 1 indicating conformity to a normal distribution.

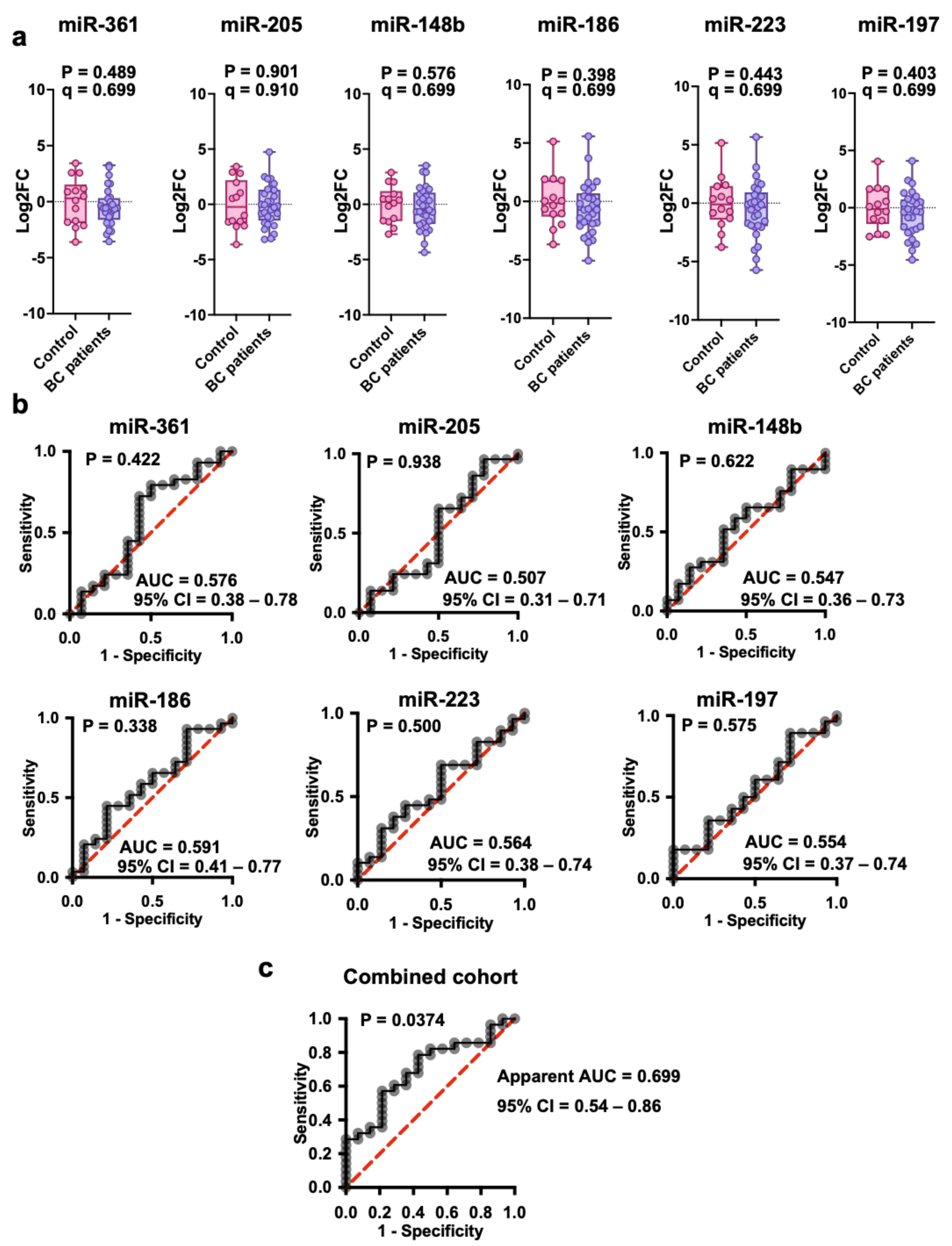

Supplementary Figure S5. Candidate circulating microRNA (c-miRNA) expression in a validation cohort of control and BC patients normalized to spike-in cel-mir-39. (a) RT-qPCR validation of selected c-miRNA candidates identified as differentially expressed between equine and canine MDEC-derived conditioned media (Fig. 3). Expression levels were normalized to the cel-miR-39 control and reported as Log2FC values, calculated using the $2^{-\Delta\Delta Ct}$ method. Each data point corresponds to an individual control or BC patient sample. Statistical significance was assessed using an unpaired Welch's *t*-test ($p < 0.05$). Adjusted p-values (q-values) were performed using the Two-stage step-up (Benjamini, Krieger, and Yekutieli) method. **(b)** Receiver operating characteristic (ROC) analysis of the candidate c-miRNAs comparing control and BC patient groups. Each curve displays the corresponding p-value, area under the curve (AUC), and 95 % CI, indicating the predictive capacity of each biomarker. n= 29 BC patients and n=14 control patients. **(c).** Multivariate analysis of all six c-miRNAs. ROC obtained via multiple logistic regression analysis. Apparent AUC and 95% CI are shown.

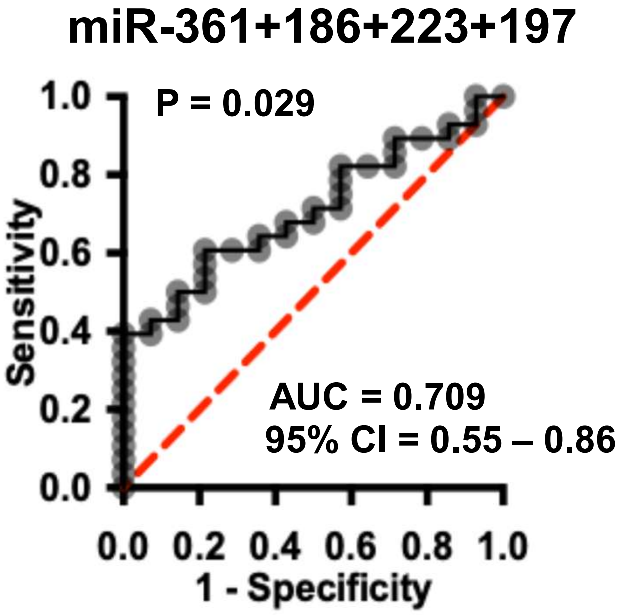

Supplementary Figure S6. Predictive capacity of filtered c-miRNA candidates normalized to RNU6. ROC curve depicting the combined discriminatory performance of miR-361, miR-186, miR-223, and miR-197, which were selected based on their univariate analysis displaying an AUC > 0.65. Multivariate model was generated using multiple logistic regression analysis to incorporate selected predictive miRNA candidates. Apparent AUC, p-value ($p < 0.05$), and 95 % CI values are shown.

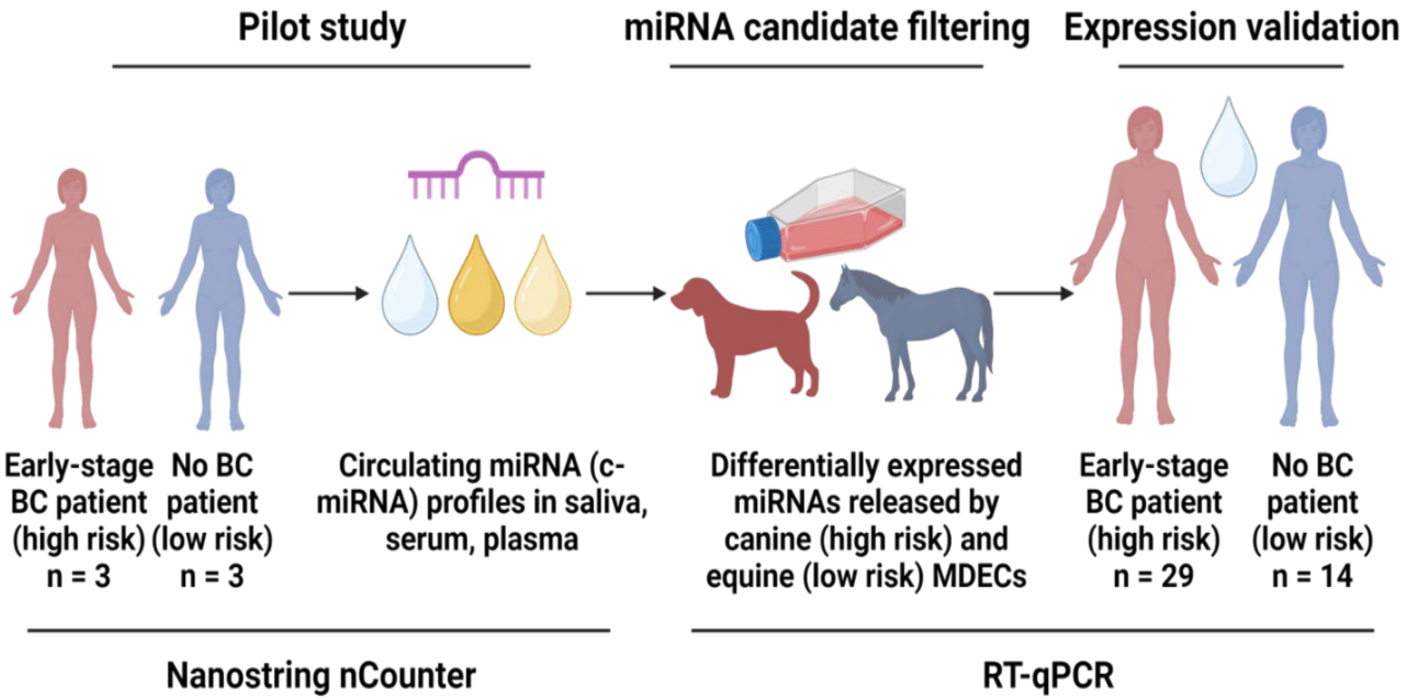

Supplementary Figure S7. Study workflow schematic for discovering circulating microRNAs (c-miRNAs) potentially associated with early breast cancer (BC) presence. First, a pilot screen (n = 3 per group) was conducted across three biofluids—saliva (light blue), serum (dark yellow), and plasma (light yellow)—using the NanoString nCounter platform. Candidate miRNAs were then filtered by measuring their secretion in conditioned media (CM) from canine and equine mammosphere-derived epithelial cells (MDECs) using reverse transcriptase (RT)-quantitative (q) PCR. Using RT-qPCR, the resulting shortlist of c-miRNAs potentially associated with early BC presence was subsequently tested in a larger patient cohort, validating expression in saliva (light blue) from BC patient (high risk) and control (No BC patient, low risk) groups.

Nanostring nCounter miRNA analysis (Patient Plasma - Breast Cancer patients [n = 3] versus Control patients (noBC) [n = 3])

miRNA	Fold change	p-value	p-Adj
hsa-let-7i-5p	-4.32033	0.26860	0.99152
hsa-miR-30b-5p	-2.12836	0.09966	0.99152
hsa-miR-374b-5p	-1.98512	0.10493	0.99152
hsa-miR-125a-5p	-1.93179	0.18405	0.99152
hsa-miR-374a-5p	-1.77008	0.18136	0.99152
hsa-miR-26a-5p	-1.72369	0.22842	0.99152
hsa-miR-29b-3p	-1.71256	0.22910	0.99152
hsa-let-7f-5p	-1.62226	0.24563	0.99152
hsa-miR-4454+hsa-miR-7975	-1.60554	0.26959	0.99152
hsa-let-7a-5p	-1.60345	0.26659	0.99152
hsa-miR-181a-5p	-1.58496	0.29255	0.99152
hsa-miR-223-3p	-1.57398	0.25634	0.99152
hsa-miR-98-5p	-1.55847	0.28653	0.99152
hsa-miR-21-5p	-1.54593	0.33322	0.99152
hsa-miR-361-5p	-1.47971	0.30685	0.99152
hsa-miR-148b-3p	-1.47622	0.36100	0.99152
hsa-let-7d-5p	-1.47498	0.36332	0.99152
hsa-miR-382-5p	-1.47179	0.36619	0.99152
hsa-miR-15b-5p	-1.47142	0.37515	0.99152
hsa-miR-107	-1.47122	0.38741	0.99152
hsa-miR-15a-5p	-1.42151	0.41888	0.99152
hsa-miR-148a-3p	-1.40179	0.43147	0.99152
hsa-miR-142-3p	-1.37552	0.42793	0.99152
hsa-miR-28-5p	-1.36418	0.40775	0.99152
hsa-miR-126-3p	-1.35608	0.47160	0.99152
hsa-miR-337-5p	-1.35076	0.47108	0.99152
hsa-miR-191-5p	-1.3427	0.46709	0.99152
hsa-miR-423-5p	-1.34031	0.50349	0.99152
hsa-miR-1260b	-1.33912	0.51492	0.99152
hsa-let-7g-5p	-1.31962	0.54166	0.99152
hsa-miR-23a-3p	-1.30019	0.52122	0.99152
hsa-miR-144-3p	-1.28691	0.62193	0.99152
hsa-miR-199a-5p	-1.27717	0.51068	0.99152
hsa-miR-340-5p	-1.27453	0.55921	0.99152
hsa-miR-29c-3p	-1.26783	0.57625	0.99152
hsa-miR-483-3p	-1.26506	0.62892	0.99152
hsa-miR-1253	-1.26379	0.51683	0.99152
hsa-miR-302d-3p	-1.22735	0.57868	0.99152
hsa-miR-146a-5p	-1.22027	0.61996	0.99152
hsa-miR-26b-5p	-1.20941	0.68870	0.99152
hsa-miR-199a-3p+hsa-miR-199b-3p	-1.18804	0.65695	0.99152
hsa-miR-302b-3p	-1.16954	0.76926	0.99152
hsa-miR-19b-3p	-1.1609	0.72217	0.99152
hsa-miR-122-5p	-1.1583	0.74802	0.99152
hsa-miR-1246	-1.15543	0.71664	0.99152
hsa-miR-130a-3p	-1.13636	0.75408	0.99152
hsa-miR-1290	-1.13119	0.74083	0.99152
hsa-miR-30d-5p	-1.1232	0.78501	0.99152
hsa-miR-185-5p	-1.10314	0.80958	0.99152
hsa-miR-320e	-1.08745	0.86254	0.99152
hsa-miR-644a	-1.08669	0.82305	0.99152
hsa-miR-106a-5p+hsa-miR-17-5p	-1.08034	0.86733	0.99152
hsa-miR-20a-5p+hsa-miR-20b-5p	-1.07168	0.87763	0.99152
hsa-miR-30e-5p	-1.05985	0.87350	0.99152
hsa-miR-25-3p	-1.00498	0.99152	0.99152
hsa-miR-22-3p	1.00824	0.98583	0.99152
hsa-miR-548aa+hsa-miR-548t-3p	1.01053	0.97637	0.99152
hsa-miR-106b-5p	1.01946	0.96628	0.99152
hsa-miR-150-5p	1.02287	0.95753	0.99152
hsa-miR-93-5p	1.0267	0.95129	0.99152
hsa-let-7b-5p	1.0299	0.95186	0.99152
hsa-miR-4455	1.03443	0.92479	0.99152
hsa-miR-221-3p	1.0599	0.88361	0.99152

hsa-miR-24-3p	1.10437	0.79332	0.99152
hsa-miR-27b-3p	1.15116	0.71209	0.99152
hsa-miR-1910-5p	1.16218	0.74169	0.99152
hsa-miR-92a-3p	1.16822	0.70614	0.99152
hsa-miR-16-5p	1.1951	0.71033	0.99152
hsa-miR-548n	1.26317	0.51523	0.99152
hsa-let-7c-5p	1.38999	0.44480	0.99152

Patient Number	Group	Age	ER status	PR status	HER2 status	Tumor Grade	Pre-surgical treatments	Clinical description of breast cancer pathology	Genetic Tests
**Breast Cancer Patients**									
1	breast cancer	59	Pos	Neg	Pos	1	No pre-surgery treatments	Left breast: invasive ductal carcinoma with tubular and cribriform features, ER-positive, PgR-negative, HER2-positive (amplified). Overall Grade: Grade 1 (scores of 3, 4 or 5). Right breast: benign fibrofatty breast tissue with patchy florid ductal hyperplasia, sclerosing adenosis, papilloma, columnar cell change, papillary apocrine change, and microcalcifications. No malignancy identified.	Peripheral blood. Test: Invitae Breast Cancer STAT panel with ATM and CHEK2 gene add ons (56 genes including BRCA1/2). Results: No pathogenic vairants or genetic variants identified. No molecular evidence for hereditary predisposition for breast/ovarian or other cancer syndromes.
2	breast cancer	41	Pos	Pos	Pos	2	No pre-surgery treatments	right breast: invasive carcinoma of no special type (ductal), ER-positive, PgR-positive, HER2: equivocal (score 2+). Overall Grade: Grade 2 (scores of 6 or 7). Left breast: benign fibrocystic changes with sclerosing adenosis and papillary apocrine metaplasia. No atypia or malignancy identified.	Peripheral blood. Test: BRCA1/2 analyses with CancerNext-expanded +RNA insight (77 genes including BCCA1/2). Results: No pathogenic mutations, variants of unknown significance, or gross deletions or duplications were detected. No clinically relevant aberrant RNA transcripts were detected in select analyzed genes.
3	breast cancer	47	Pos	Pos	Neg	1	No pre-surgery treatments	no surgery, clinic visit, pathology from biopsy: invasive ductal carcinoma with tubular features, grade 1, ER-positive, PgR-positive, HER2-negative	Test: Invitae 84-gene multi-cancer panel including BRCA1/2 genes. Results: (a.) One Increased Risk Allele in APC at locus c.3920T>A [a/k/a p. Ile 1307 Lys]; (b.) VARIANT OF UNDETERMINED SIGNIFICANCE in: POLE at locus c.882G>A [a/k/a p.Met 294 Ile]. INTERPRETATION: Increased risk of colorectal cancer, but not as high as that with true Familial Adenomatous Polyposis. No other molecular evidence of other hereditary cancer syndromes including breast/ovarian, other gastrointestinal, urinary, endocrine cancers, or melanoma.
4	breast cancer	78	Pos	Pos	Neg	1	No pre-surgery treatments	invasive carcinoma of no special type (ductal), ER-positive, PgR-positive, HER2-negative. Overall Grade: Grade 1 (scores of 3, 4 or 5)	Peripheral blood. Test: Invitae Common Hereditary Cancers Panel, Invitae Breast Cancer Panel, Add on preliminary -evidence genes for breast cancer, Invitae breast cancer STAT panel, add on ATM gene, add on CHEK2 gene. 56 genes analyzed including BRCA1/2. Results: No pathogenic variants identified. Negative for any high-risk mutations in high-risk breast cancer genes. Negative for any deleterious mutation in the gene panel. Variant of undetermined significance indentified in SDHA gene: SDHA c.5C>T (p.Ser2Leu) heterozygous Uncertain Significance. No molecular evidence for hereditary preidsposition for breast/ovarian or other cancer syndromes. The clinical significance of the variant(s) identified in this gene is uncertain.
5	breast cancer	50	Pos	Pos	Neg	1	No pre-surgery treatments	invasive carcinoma of no special type (ductal). Ductal carcinoma in situ. Biopsy site changes. ER positive, PR positive, HER-2 negative. Overall Grade: Grade 1 (scores of 3, 4 or 5)	Peripheral blood. Test: BRCA1/2 analyses with CancerNext +RNAinsight (36 genes including BRCA1/2). Results: No pathogenic mutations, variants of unknown significance, or gross deletions or duplications were detected. No clinically relevant aberrant RNA transcripts were detected in select analyzed genes. No molecular evidence for hereditary predisposition to breast/ovarian or other cancer syndromes.
6	breast cancer	65	Pos	Pos	Neg	1	No pre-surgery treatments	invasive carcinoma of no special type (ductal). Features of tubular and cribriform carcinoma are present. ER-positive, PgR: positive, HER2: negative. Overall grade: Grade 1 (scores of 3, 4 or 5)	Peripheral blood. Test: BRCA1/2 analyses with CancerNext-expanded +RNAinsight (77 genes including BRCA1/2). Results: No pathogenic mutations, variants of unknown significance, or gross deletions or duplications were detected. No clinically relevant aberrant RNA transcripts were detected in select analyzed genes. No molecular evidence for hereditary predisposition to breast/ovarian or other cancer syndromes.

7	breast cancer	51	Pos	Pos	Neg	3	No pre-surgery treatments	solid papillary carcinoma with invasion. Overall Grade: Grade 3 (scores of 8 or 9). ER-positive, PgR: positive, HER2: negative.	Peripheral blood. Test: BRCA1/2 analyses with CancerNext +RNA insight, BRCA1/2 analyses with BRCAplus , gene sequence and deletion/duplication analyses of BRCA1 and BRCA2 (36 genes analyzed including BRCA1/2). Result: No pathogenic mutations, variants of unknown significance, or gross deletions or duplications were detected. No clinically relevant aberrant RNA transcripts were detected. No molecular evidence for hereditary predisposition to breast/ovarian or other cancer syndromes.
8	breast cancer	64	Pos	Pos	Neg	2	Was on HRT prior to dianosis. No pre-surgery treatments	invasive carcinoma of no special type (ductal). ER-positive; PgR-positive; HER2-negative. Overall Grade: Grade 2 (scores of 6 or 7). All regional lymph nodes negative for tumor. 0/1 lymph nodes involved.	Peripheral blood. Test: Invitae sequence analysis and deletion/duplication testing for 84 genes, including invitae multi-cancer panel, including BRCA1/2. Results: negative, the test did not identify any pathogenic variants known to cause disease. No reportable genetic variants were identified.
9	breast cancer	60	Pos	Pos	Neg	N/A	Was on HRT prior to dianosis. No pre-surgery treatments	invasive ductal carcinoma. ER-positive, PgR-positive, HER2-negative.	Peripheal blood sample. Test: BRCA1/2 analyses with CancerNext-expanded +RNA insight (77 genes including BRCA1/2), BRCA1/2 analyses with BRCAplus (8 genes including BRCA1/2). Results: No pathogenic mutations, variants of unknown significance, or gross deletions or duplications detected. No clinically aberrant RNA transcripts were detected. No molecular evidence for hereditary preidsposition for breast/ovarian or other cancer syndromes.
10	breast cancer	41	Pos	Pos	Neg	2	No pre-surgery treatments	multifocal invasive ductal carcinoma. Ductal carcinoma in situ. Pseudoangiomatous stromal hyperplasia. Number of Foci: 2. -ER-pos, PR-pos, HER2-neg. Overall Grade: Grade 2 (scores of 6 or 7)	Peripheral blood sample: Test: BRCA1/2 analyses with CancerNext +RNAinsight, gene sequence and deletion/duplication analyses of BRCA1/2, and BRCA1/2 analyses with BRCAplus (36 genes analyzed including BRCA1/2). Results: No pathogenic mutations, variants of unknown significance, or gross deletions or duplications detected. No clinically aberrant RNA transcripts were detected. No molecular evidence for hereditary preidsposition for breast/ovarian or other cancer syndromes.
11	breast cancer	69	Pos	Pos	Pos	1	No pre-surgery treatments	invasive carcinoma of no special type (ductal). ER-positive, PgR: positive, HER2: positive. Overall Grade: Grade 1 (scores of 3, 4 or 5).	Peripheral blood sample: Test: BRCA1/2 analyses with BRCAplus, CancerNext +RNAinsight and gene sequence and deletion /duplication BCRA1/2 analysis with CancerNext +RNAinsight (36 genes analyzed including BRCA1/2). Results: No pathogenic mutations, variants of unknown significance, or gross deletions or duplications detected. No clinically aberrant RNA transcripts were detected. No molecular evidence for hereditary preidsposition for breast/ovarian or other cancer syndromes.
12	breast cancer	50	Pos	Neg	Neg	1	No pre-surgery treatments	left breast: ductal carcinoma in situ. ER-positive, PR-negative, HER2-negative. Right breast: benign fibrofatty breast tissue with adenosis, columnar cell change, fibroadenomatoid change, papillary apocrine change, focal mild ductal hyperplasia and microcalcifications. No malignancy identifed.	Test: Invitae breast cancer panel and Invitae Multi-cancer panel (84 genes including BRCA1/2). Results: Negative for any deleterious mutations. No molecular evidence for hereditary predisposition for breast/ovarian or other cancer syndromes.
13	breast cancer	65	Pos	Pos	Neg	1	No pre-surgery treatments	invasive carcinoma of no special type (ductal), ER-positive, PgR-positive, HER2-negative, Overall Grade: Grade 1 (scores of 3, 4 or 5)	Peripheral blood. Invitae multi cancer and breast cancer STAT panels with ATM and CHEK2 gene add ons (93 genes incluidng BRCA1/2). Results: No pathogenic variants or genetic variants identified. No molecular evidence for hereditary predisposition for breast/ovarian or other cancer syndromes.
14	breast cancer	76	1	1	N/A	3	No pre-surgery treatments	ductal carcinoma in situ, ER-positive, PgR-positive, nuclear Grade: Grade III (high)	Subject declined genetic testing.

15	breast cancer	68	1	1	1	2	No pre-surgery treatments	invasive carcinoma of no special type (ductal), ER-positive, PgR-positive, HER2: positive (amplified), Overall Grade: Grade 2 (scores of 6 or 7)	Peripheral blood. Test: Invitae Common hereditary cancers panel and breast Cancer STAT panel with ATM and CHEK2 gene add ons (56 genes including BRCA1/2). Results: No pathogenic variants or genetic variants identified. No molecular evidence for hereditary predisposition for breast/ovarian or other cancer syndromes.
16	breast cancer	65	Pos	Pos	Neg	2	No pre-surgery treatments	Pathology from prior biopsy and 1st surgery (partial mastectomy): microinvasive ductal carcinoma plus large areas of DCIS, grade 2, ER/PR positive, HER2 negative. Pathology from current surgery. breast, left: extensive biopsy site changes with foreign body giant cell reaction and fibrosis. Surrounding breast parenchyma with fibrocystic changes, adenosis, focal intraductal papilloma and usual ductal hyperplasia. No malignancy identified. breast tissue, right: benign breast parenchyma, focal adenosis and usual ductal hyperplasia.	Test: Invitae Common Hereditary Cancer Panel and Invitae Breast Cancer STAT Panel, add on ATM gene, add on CHEK2 gene (47 gene panel including BRCA1/2). Results: No pathogenic variants or genetic variants identified. No molecular evidence for hereditary predisposition for breast/ovarian or other cancer syndromes.
17	breast cancer	57	Pos	Pos	Neg	2	No pre-surgery treatments	invasive carcinoma of no special type (ductal). Overall Grade: Grade 2 (scores of 6 or 7). Single focus of invasive carcinoma. ER-positive, PgR-positive, HER2-negative.	Peripheral blood sample, Test: BRCA1/2 analyses with CancerNext-Expanded +RNAinsight (77 genes including BRCA 1/2), Results: No pathogenic mutations, variants of unknown significance, or gross deletions/duplications were detected. No molecular evidence for hereditary predisposition for breast/ovarian or other cancer syndromes.
18	breast cancer	50	Pos	Pos	N/A	2	No pre-surgery treatments	Breast, left: small foci of ductal carcinoma in situ and small focus of invasive ductal carcinoma. Invasive carcinoma of no special type (ductal). Overall Grade: Grade 2 (scores of 6 or 7). ER-positive, PgR-positive, HER2 (pending). Breast, right: fibrofatty breast tissue with florid ductal hyperplasia, papillomas, sclerosing adenosis, fibroadenomatoid change, columnar cell change, apocrine metaplasia and cystic change, prominent microcalcifications.	Peripheral blood sample, Test: BRCA1/2 analyses with CancerNext-Expanded +RNAinsight (77 genes including BRCA 1/2), Results: This individual is heterozygous for the p.L862V (c.2584T>G) variant of unknown significance in the CDH1 gene and heterozygous for the p.F245S (c.734T>C) variant of unknown significance in the SUFU gene, which may or may not contribute to this individual's clinical history. However, the clinical significance of the locus c.734T>C (p.F245S) in the SUFU gene is uncertain at this time. In summary, the available evidence is currently insufficient to determine the role of this variant in disease. Therefore, this SUFU gene mutation has been classified as a Variant of Uncertain Significance (VUS). At this time, this result is insufficient to cause SUFU-related disease.
19	breast cancer	52	Pos	Pos	Neg	1	No pre-surgery treatments	**Breast, left:** invasive carcinoma with features of tubular and cribriform carcinoma. Overall Grade: Grade 1 (scores of 3, 4 or 5). ER-positive, PgR-positive, HER2-negative. **Breast, right:** invasive carcinoma with features of tubular carcinoma. Overall Grade: Grade 1 (scores of 3, 4 or 5). ER-positive; PgR-positive; HER2-negative.	Peripheral blood sample, Test: BRCA1/2 analyses with CancerNext-Expanded +RNAinsight (77 genes including BRCA 1/2), Results: No pathogenic mutations, variants of unknown significance, or gross deletions/duplications were detected.
20	breast cancer	72	Pos	Pos	Neg	1	No pre-surgery treatments	Right breast: Invasive carcinoma of no special type (ductal). Overall Grade: Grade 1 (scores of 3, 4 or 5). ER-positive, PgR-positive, HER2-negative. Left breast: Invasive carcinoma of no special type (ductal). Histologic Type Comment: Focal micropapillary features. Overall Grade: Grade 2 (scores of 6 or 7). ER-positive, PgR-positive, HER2-negative.	Peripheral blood sample: Test: BRCA1/2 Analyses with Cancernext-Expanded +RNAinsight (77 genes including BCCA1/2), Results: One variant of unknown significance was detected in the POT1 gene. Individual is heterozygous for the p.V326L (c.976G>C) variant of unknown significance in the POT1 gene. Interpretation: no molecular evidence for hereditary predisposition for breast/ovarian or other cancer syndromes.
21	breast cancer	59	Pos	Pos	Neg	2	No pre-surgery treatments	invasive ductal carcinoma in the background of extensive ductal carcinoma in situ. Invasive carcinoma of no special type (ductal). Overall Grade: Grade 2 (scores of 6 or 7). ER-positive; PgR-positive; HER2-negative.	Peripheral blood sample, Test: BRCA1/2 analyses with CancerNext-Expanded +RNAinsight (77 genes including BRCA 1/2), Results: No pathogenic mutations, variants of unknown significance, or gross deletions/duplications were detected.
22	breast cancer	72	Pos	Pos	Neg	1	No pre-surgery treatments	invasive carcinoma of no special type (ductal). Overall Grade: Grade 1 (scores of 3, 4 or 5). ER-positive; PgR-positive; HER2-negative	Subject declined genetic testing.

23	breast cancer	65	Pos	Pos	Neg	1	No pre-surgery treatments	invasive carcinoma of no special type (ductal). Overall Grade: Grade 1 (scores of 3, 4 or 5). ER+/PR+/HER2-. Tumor present in regional lymph node(s). 1/1 sentinel lymph nodes involved.	Peripheal blood sample, Test: Multi-Cancer + RNA Panel, Multi-Cancer Genes Eligible for RNA Analysis, Breast Cancer STAT Panel, Add-On ATM Gene, and Add-On CHEK2 Gene (84 genes including BRCA 1/2). No pathogenic variants or mutations were detected.
24	breast cancer	64	Pos	Pos	Neg	2	No pre-surgery treatments	Invasive carcinoma of no special type (ductal). ER+/PR+/HER2- . Overall Grade: Grade 2 (scores of 6 or 7).	Peripheral blood sample, Test: BRCA1/2 analyses with CancerNext-Expanded +RNAinsight (77 genes including BRCA 1/2), Results: No pathogenic mutations, variants of unknown significance, or gross deletions/duplications were detected.
25	breast cancer	64	Pos	Pos	Neg	2	No pre-surgery treatments	invasive carcinoma of no special type (ductal). ER-positive, PgR-positive, HER2-negative. Overall Grade: Grade 2 (scores of 6 or 7).	No genetic testingi n medical record.
26	breast cancer	50	Pos	Pos	Neg	2	No pre-surgery treatments	**RIGHT BREAST:** Invasive carcinoma with mixed ductal and lobular features. Overall Grade: Grade 2 (scores of 6 or 7). ER-positive; PgR-positive; HER2-negative. **LEFT BREAST:** benign breast parenchyma with fibrocystic changes. No tumor identified.	Peripheral blood sample, Test: BRCA1/2 analyses with CancerNext-Expanded +RNAinsight (77 genes including BRCA 1/2), Results: No pathogenic mutations, variants of unknown significance, or gross deletions/duplications were detected.
27	breast cancer	71	Pos	Pos	Pos	1	No pre-surgery treatments	Invasive carcinoma of no special type (ductal).ER-positive; PgR-positive; HER2-positive. Overall Grade: Grade 1 (scores of 3, 4 or 5)	Peripheral blood sample, Test: BRCA1/2 analyses with CancerNext-Expanded +RNAinsight (77 genes including BRCA 1/2), Results: No pathogenic mutations, variants of unknown significance, or gross deletions/duplications were detected.
28	breast cancer	54	Pos	Pos	Neg	N/A	No pre-surgery treatments	LEFT breast: Ductal carcinoma in situ. ER-positive; PgR-positive; HER2-negative. RIGHT breast: flat epithelial atypia. Fibrocystic changes and microcalcifications. Fibrosis and hemorrhage consistent with prior biopsy site changes.	Peripheral blood sample, Test: BRCA1/2 analyses with CancerNext-Expanded, RNAinsight, BRCAplus (77 genes including BRCA 1/2), Results: No pathogenic mutations, variants of unknown significance, or gross deletions/duplications were detected.
29	breast cancer	71	Pos	Pos	Neg	2	No pre-surgery treatments	invasive carcinoma of no special type (ductal). Overall Grade: Grade 2 (scores of 6 or 7). ER-positive, PgR-positive, HER2-negative.	Peripheral blood sample, Test: BRCA1/2 analyses with CancerNext-Expanded +RNAinsight (77 genes including BRCA 1/2), Results: No pathogenic mutations, variants of unknown significance, or gross deletions/duplications were detected.

Patient Number	Group	Age	ER status	PR status	HER2 status	Tumor Grade	Pre-surgical treatments	Clinical reason for surgery.	Genetic Tests
**Control Patients**									
30	No cancer ctrl	61					N/A	pelvic organ prolapse	Genetic testing data was not performed/not available for no cancer ctrl patients
31	No cancer ctrl	57					N/A	pelvic organ prolapse	
32	No cancer ctrl	71					N/A	pelvic organ prolapse	
33	No cancer ctrl	71					N/A	vaginal prolapse, midline cystocele	
34	No cancer ctrl	57					N/A	female cystocele, uterus prolapse	
35	No cancer ctrl	69					N/A	prolapse of female pelvic organs	
36	No cancer ctrl	62					N/A	thyroglossal duct cyst	
37	No cancer ctrl	67					N/A	pelvic organ prolapse	
38	No cancer ctrl	65					N/A	pelvic organ prolapse	
39	No cancer ctrl	62					N/A	prolapse of female pelvic organs	
40	No cancer ctrl	70					N/A	uterine prolapse	
41	No cancer ctrl	53					N/A	vaginal prolapse	
42	No cancer ctrl	50					N/A	female cystocele, prolapse of female pelvic organs	

43	No cancer ctrl	75					N/A	cystocele with rectocele	

Supplementary Table S3. MicroRNA (miRNA) primers for RT-qPCR.

Probe Name	Mature Sequence	Assay ID*
miR-342	UCUCACACAGAAAUCGCACCCGU	002260
miR-25	CAUUGCACUUGUCUCGGUCUGA	000403
miR-23a	AUCACAUUGCCAGGGAUUUCC	000399
miR-93	AAAGUGCUGUUCGUGCAGGUAG	000432
miR-16	UAGCAGCACGUAAAUAUUGGCG	000391
miR-423	UGAGGGGCAGAGAGCGAGACUUU	002340
miR-222	AGCUACAUCUGGCUACUGGGU	002276
miR-450a	UUUUGCGAUGUGUUCCUAAUAU	002303
miR-361	UUAUCAGAAUCUCCAGGGUAC	000554
miR-205	UCCUUCAUUCCACCGGAGUCUG	000509
miR-148b	UCAGUGCACUACAGAACUUUGU	000471
miR-223	UGUCAGUUUGUCAAAUACCCCA	002295
miR-197	UUCCACCACCUUCUCCACCCAGC	000497
miR-186	CAAAGAAUCUCCUUUUGGGCU	002285
cel-miR-39	UCACCGGGUGUAAAUCAGCUUG	000200
U6 small nuclear-1 (RNU6)	GTGCTCGCTTCGGCAGCACATATACTAAAA<br>TTGGAACGATACAGAGAAGATTAGCATGGC<br>CCCTGCGCAAGGATGACACGCAAATTCGTG<br>AAGCGTTCCAATATTTT	001973

*TaqMan miRNA assays (Thermofisher Scientific, Waltham, MA)
